# An Action-Centric Zero Trust Maturity Model for Agentic AI Environments

**DOI:** 10.3390/s26165205

**Published:** 2026-08-17

**Authors:** Jung-Hyun Mok, Hyun Jo, Sokjoon Lee

**Affiliations:** 1 Department of Information Security, Gachon University, Seongnam-si 13120, Republic of Korea; johnmok@gachon.ac.kr (J.-H.M.); hbin7111@iwest.co.kr (H.J.); 2 Information Security Office, Korea Western Power, 285, Jungang-ro, Taean-eup, Taean-gun 32140, Republic of Korea; 3 Department of Smart Security, Gachon University, Seongnam-si 13120, Republic of Korea

**Keywords:** Zero Trust, AI agent security, maturity model

## Abstract

Large language model–based agentic AI systems can independently interpret user goals, develop plans, and interact with external tools. These capabilities introduce security concerns that extend beyond traditional access control. However, existing Zero Trust Maturity Models, such as the CISA ZTMM, mainly focus on how resources are accessed and provide limited guidance on how to evaluate actions taken after access has been granted. This paper proposes AI-ZTMM, which extends CISA’s five-pillar structure to action-level trust evaluation. The model defines forty-one security Functions based on ten threat categories and thirty-one security requirements and introduces Action Space and seven Action Risk Factors for organizational self-assessment. Its scope includes software agents and the software action layer of agents in IoT, robotic, and OT/ICS environments. The model was refined through reviews by eleven domain experts and evaluated using thirty-eight MITRE ATLAS case studies. The CISA ZTMM lacked directly relevant controls for 59.7% of the analyzed attack stages, whereas AI-ZTMM addressed 75.4% of this gap, achieving a combined direct coverage of 84.4%. These results show that AI-ZTMM complements the CISA ZTMM by providing action-level security controls.

## 1. Introduction

Agentic AI, built on large language models, extends generative AI from response generation to autonomous action execution. It can interpret goals, plan tasks, invoke tools and APIs, retrieve external data, and delegate tasks to other agents [1,2]. Although these capabilities improve automation, they also create new attack surfaces because agent outputs may directly trigger executable actions in external systems [1,3,4].

Zero Trust continuously verifies subjects, resources, and access requests rather than implicitly trusting entities within a network boundary. The CISA Zero Trust Maturity Model (ZTMM) provides a representative organizational framework based on five Pillars: Identity, Devices, Networks, Applications and Workloads, and Data [5]. However, it primarily evaluates resource access and does not treat autonomous agent actions, such as tool invocation, privilege delegation, memory use, and external system interaction, as independent assessment targets.

Recent studies have proposed security controls, authorization mechanisms, autonomy tiers, and governance frameworks for Agentic AI [2,6,7,8,9,10,11,12,13,14]. However, no model was identified in the literature reviewed that preserves the CISA five-Pillar structure while assessing agent actions as an independent unit and supporting Pillar-level organizational self-assessment.

To address this gap, this paper proposes the Agentic AI Zero Trust Maturity Model (AI-ZTMM), an action-centric maturity model for software-based Agentic AI systems. AI-ZTMM retains the CISA five-Pillar structure while extending trust evaluation from access to action. It introduces Action Space to define the range of actions available to an agent and Action Risk Factors to assess the risks associated with those actions. The model also covers agents deployed in IoT, robotic, and OT/ICS environments, while limiting its assessment to the software action layer, including goal interpretation, task planning, tool invocation, memory and context handling, and control-command generation.

The main contributions of this paper are threefold. First, it redefines Pillar-specific evaluation criteria for Agentic AI while preserving the CISA five-Pillar structure. Second, it extends Zero Trust maturity assessment from access-centric to action-centric control through Action Space and Action Risk Factors. Third, it provides qualitative maturity criteria that organizations can use to assess control levels and identify security gaps.

The remainder of this paper is organized as follows. Section 2 reviews related concepts and prior studies. Section 3 describes the research design and requirement-derivation process. Section 4 presents AI-ZTMM and its maturity criteria. Section 5 evaluates the model using traceability analysis and MITRE ATLAS cases. Section 6 and Section 7 discuss limitations, future work, and conclusions.

## 2. Background and Related Work

### 2.1. Zero Trust Maturity Models

The concept of Zero Trust gained prominence in security architecture discussions in 2010, when John Kindervag of Forrester Research proposed the Zero Trust network model to address the limitations of conventional perimeter-based security [15]. NIST later defined Zero Trust in SP 800-207 as an architecture in which no entity is implicitly trusted based on its network location and access is granted only after authentication, authorization, and communication protection [16].

CISA operationalized these principles through the Zero Trust Maturity Model (ZTMM), which supports the staged adoption of Zero Trust. The model comprises five Pillars—Identity, Devices, Networks, Applications and Workloads, and Data—and three Cross-cutting Capabilities: Visibility and Analytics, Automation and Orchestration, and Governance [5]. It also defines four maturity levels—Traditional, Initial, Advanced, and Optimal—through which organizations can assess their security posture and identify areas for improvement.

**Figure 1:**
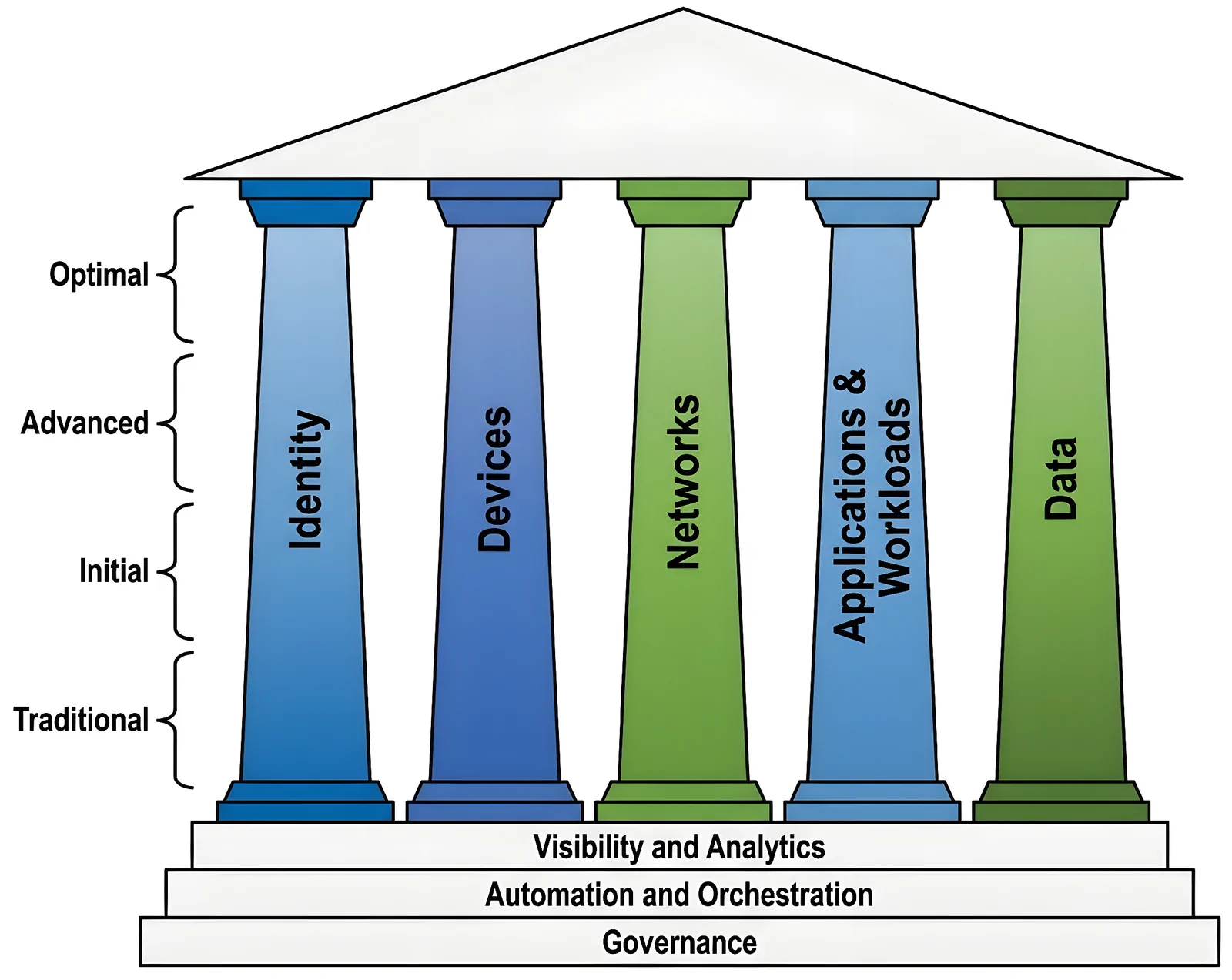
CISA Zero Trust Pillars and Cross-cutting Capabilities [5].

However, existing Zero Trust maturity models primarily focus on resource access and operational controls related to users, devices, networks, applications, and data. Consequently, they do not adequately assess the autonomous actions of AI agents that interpret goals, invoke tools, and interact with external systems. To address this limitation, this paper retains the CISA five-Pillar structure while redefining the assessment targets and criteria of each Pillar for Agentic AI environments. Figure 1 shows the structure of the CISA ZTMM.

### 2.2. Security Characteristics of Agentic AI

Agentic AI exhibits several security characteristics that distinguish it from conventional LLM applications. Its execution path may vary according to runtime inputs, external context, and tool responses, making multi-step behavior difficult to predict [8]. Through tools and APIs, agents can perform not only read operations but also state-changing actions, including writing, deletion, and execution [4,17]. In orchestrator–sub-agent structures, tasks and privileges may be dynamically delegated, resulting in fluid rather than fixed trust boundaries [18,19]. Moreover, the integrity of memory and retrieval-augmented generation stores directly influences an agent’s decisions and action selection [20]. Integration with external tools, MCP servers, plugins, and other agents further expands the attack surface [4,10].

These characteristics indicate that Agentic AI security requires system-level action control in addition to model-level robustness. Christodorescu et al. argue that agent security should be treated as a systems problem: the underlying AI model should be regarded as an untrusted component, while security invariants should be enforced at the system level rather than relying solely on model safety [21]. Public threat taxonomies similarly show that interactions among inputs, memory, tools, APIs, and external services can lead to goal manipulation, tool misuse, privilege abuse, and information disclosure [1,22].

Accordingly, agents with identical privileges may present different levels of risk depending on their goals, tool sequences, reversibility, data sensitivity, delegation structures, and autonomy levels. Security control should therefore extend beyond access verification to action verification. Section 4 operationalizes this perspective through Action Space, Action Risk Factors, and Pillar-specific Functions.

### 2.3. Threat Landscape and Zero Trust Guidance for AI Agents

The security characteristics of Agentic AI expand the attack surface beyond that of conventional LLM applications. Public threat taxonomies have systematically categorized these risks. In addition to the OWASP Top 10 for LLM Applications [23], OWASP identified seventeen Agentic AI-specific threats, including memory poisoning, tool misuse, privilege compromise, goal manipulation, inter-agent communication poisoning, and supply chain compromise [22]. The OWASP Agentic Top 10 further consolidates these threats into ten high-risk categories, including Agent Goal Hijack, Tool Misuse, Identity and Privilege Abuse, and Memory and Context Poisoning, to support practitioner-oriented prioritization [1]. CISA-led joint guidance similarly classifies Agentic AI risks into privilege, design and configuration, behavior, structural, and accountability risks, while providing lifecycle guidance from design to operation [13].

Several industry frameworks have applied Zero Trust principles to address these threats. Anthropic proposes a Zero Trust security framework for autonomous agents and defines capability tiers according to agent autonomy, based on the principles of “never trust, always verify,” “assume breach,” and “least privilege and least agency” [2]. The Cloud Security Alliance’s Agentic Trust Framework treats agents as “digital employees” and presents governance maturity stages in which autonomy is granted according to demonstrated trustworthiness [14]. Conceptual architecture studies have also applied Zero Trust principles to LLM and autonomous agent environments [8,24]. Although these frameworks provide agent-centric trust and maturity models, they are not directly aligned with the CISA five-Pillar structure.

### 2.4. Academic Approaches to Agent Security and Risk Assessment

Academic research on Agentic AI security has developed along three main directions: threat analysis, individual security controls, and risk assessment. Prior studies show that agent autonomy, tool use, external integration, and multi-agent interaction expand the attack surface beyond vulnerabilities in a single model [3,4,11,12]. Specific attack vectors, including tool impersonation and logic-layer threats in the Agentic Web, further demonstrate that Agentic AI risks arise from interactions among system components [12,25]. Chhabra et al. similarly argue that agentic risk differs from both traditional AI safety and conventional software security because planning, tool use, memory, and autonomy jointly produce system-level effects [3].

Studies on individual controls have proposed mechanisms for agent identity and delegation [6,18], tool execution and runtime control [17,26,27], memory and RAG integrity [20], and multi-agent or external interaction security [10,19,28]. Related work has also examined auditable workflows, enterprise AI security architectures, and domain-specific Zero Trust architectures [24,29,30]. In parallel, risk assessment frameworks for LLM-based multi-agent systems and Agentic AI have considered autonomy and action risk as assessment factors [9,31].

A separate research stream focuses on protocols for discovering and invoking external tools, particularly the Model Context Protocol (MCP). Tool squatting has been identified as a protocol-level threat and addressed through registry-based controls [25]. Empirical studies of public MCP registries also report weak registration vetting and show that attacker-controlled tool metadata can influence model reasoning without exploiting code-level vulnerabilities [32,33]. These findings indicate that security controls are required not only within agents but also at the points where tools are registered, selected, and invoked.

Overall, existing studies provide valuable insights into Agentic AI threats, controls, architectures, and risk factors. However, they do not primarily assess the maturity of organizational controls over agent-specific actions, such as tool invocation, privilege delegation, and memory use.

### 2.5. Research Gap

Prior studies can be grouped into three streams. First, organization-level Zero Trust maturity models based on the CISA ZTMM provide staged criteria for assessing existing IT security postures but do not explicitly treat autonomous agents as assessment targets [5]. Second, studies on Zero Trust controls and architectures for Agentic AI propose mechanisms for identity, delegation, tool execution, and memory integrity [6,18,25,30,34]. However, these studies are not organized as organization-level maturity assessment criteria [8,17,24,26,28,29]. Third, agent-centric trust-stage models and risk assessment frameworks consider autonomy and action risk [21,27], but they remain only loosely aligned with the CISA five-Pillar maturity structure.

Table 1 compares these approaches using four requirements: a maturity assessment structure, alignment with the CISA five Pillars, consideration of agent action risks, and support for organizational self-assessment. Existing approaches satisfy only some of these requirements. Although Agentic AI security controls and risk assessment have been actively studied, a maturity model that integrates action-level security requirements with the CISA structure remains underdeveloped. To address this gap, this paper retains the CISA five-Pillar structure and translates Agentic AI action-security requirements into Pillar-specific maturity criteria.

## 3. Research Methodology

### 3.1. Research Design and Scope

This paper adopts a literature-based design and analytical approach to develop a Zero Trust maturity assessment model for Agentic AI environments. The research procedure identifies major threat areas from public taxonomies, derives security requirements from relevant guidelines and prior studies, and maps them to the CISA Pillars and Cross-cutting Capabilities using a predefined codebook (File S1). The mapping results are subsequently refined into Pillar-specific Functions and maturity criteria. Throughout this process, successive versions of the model were reviewed and refined through the expert review sessions described in Section 3.4.

### 3.2. Literature Selection

Academic publications and official documents were selected separately due to their different roles in model development. Academic sources were used to derive threats, controls, and assessment concepts, while official documents and frameworks provided structural and normative foundations. This study is design-oriented rather than a systematic review; therefore, the literature was used to support specific model components rather than to comprehensively map the field.

Academic publications from January 2025 to June 2026 were considered, reflecting the emergence of agent-specific threat taxonomies in 2025 [1,22]. Candidate studies were identified through title-based searches in Google Scholar by combining agent-related terms (“agentic AI,” “AI agent,” “LLM agent,” “autonomous agent,” and “multi-agent”) with security-related terms (“zero trust,” “security,” “securing,” and “threat”). The search was supplemented by backward and forward citation tracking of the retrieved studies. Eligible publication types included English-language journal articles, conference papers, and preprints. A study was included if it addressed agentic systems (e.g., autonomy, tool use, delegation, or memory) and contributed to threat analysis, control design, or Zero Trust–related assessment. Studies on general LLMs, non-system-level robustness, non-LLM multi-agent systems, or non-primary sources were excluded.

Selection was purposive: sources were retained when they supported a specific element of the threat–requirement–function chain or served as a reference point. This ensures traceability of model construction without claiming exhaustiveness. Official documents and frameworks were selected separately based on relevance to structure, requirements, and comparison criteria [1,2,5,13,14,22,23]. The final dataset consists of 23 academic publications and seven official documents.

### 3.3. Requirement Extraction and Codebook Development

To connect threats and security requirements to maturity evaluation criteria, this paper designed a codebook for consistently mapping control items extracted from the literature to the CISA ZTMM structure. The extraction unit for requirements was defined as the minimum control unit, such as an individual control, mitigation measure, or security principle presented in the literature. Semantically equivalent controls across different sources were consolidated into a single requirement. Each requirement was documented with its source, corresponding threat, control objective, and mapping rationale. Requirements were included only when they were specific to Agentic AI or required control methods different from conventional LLM or IT environments.

The codebook was used as a classification criterion for assigning literature-derived security requirements to the Pillars and Cross-cutting Capabilities of AI-ZTMM. Each entry consists of the classification target, Agentic AI perspective, assignment criterion, boundary decision criterion, representative Functions, corresponding threats, and supporting references. This reduces subjective variation in assigning requirements to Pillars.

The extraction of requirements and their assignment to Pillars were carried out by a single coder applying this codebook. The resulting requirement set and Pillar assignments were instead submitted to the expert panel described in Section 3.4, whose qualitative feedback was used to consolidate overlapping requirements, separate control objectives that had been conflated, and resolve cases lying near a Pillar boundary.

### 3.4. Expert Review

To assess the logical consistency and practical applicability of the proposed model, review sessions were conducted with domain experts. The sessions followed the group-based approach to artifact refinement and evaluation used in design science research [35]: experts examine successive versions of the artifact, and their comments are used to revise it. The panel consisted of eleven information security experts from academia, government-funded research institutes, quasi-governmental agencies, and the private sector. It was formed to incorporate both academic perspectives on Zero Trust and AI security and operational perspectives from organizations responsible for securing public infrastructure and enterprise environments. Table 2 summarizes the composition of the expert panel.

Six in-person sessions were conducted at monthly intervals from January to June 2026, with each session lasting approximately two hours. Before each session, the latest version of the model was distributed to the panel for review. Each session began with a presentation of the agenda, followed by the panelists’ comments and group discussion. The authors reviewed the points on which the panel had reached general agreement and incorporated them into the model. The revised model was then presented at the subsequent session for further review. This process formed an iterative refinement cycle, with the agenda for each session informed by the outcomes of the preceding session.

The expert review identified practical issues that were not apparent from the literature alone. The feedback informed the selection of the threat-classification basis, the consolidation and separation of security requirements, and the wording of the maturity criteria. Consensus was reached through repeated discussion and qualitative convergence rather than through formal voting or quantitative rating.

## 4. Proposed AI Zero Trust Maturity Model

### 4.1. Overview of AI-ZTMM

The Agentic AI Zero Trust Maturity Model (AI-ZTMM) is a qualitative maturity assessment model that extends the CISA Zero Trust Maturity Model to address security requirements arising from the autonomous actions of Agentic AI systems. It enables organizations to assess their level of security control across identity, execution, communication, application, and data domains and to identify priorities for improvement.

AI-ZTMM retains the five Pillars of the CISA ZTMM—Identity, Devices, Networks, Applications and Workloads, and Data—as well as the three Cross-cutting Capabilities of Visibility and Analytics, Automation and Orchestration, and Governance. The model also introduces Action Space and Action Risk Factors to assess agent actions, including tool invocation, data processing, external system interaction, privilege delegation, reversibility, privilege level, and potential external impact. Each Pillar is assessed across four maturity levels: Traditional, Initial, Advanced, and Optimal.

The evaluation criteria were developed through the threat-based requirement derivation and codebook-based mapping procedures described in Section 3. Security requirements derived from Agentic AI threats were subsequently refined into Pillar-specific Functions and maturity-level criteria. Based on this structure, this section presents the design principles of AI-ZTMM, the concepts of Action Space and Action Risk Factors, and the evaluation criteria for each Pillar.

### 4.2. Design Principles

AI-ZTMM is based on three design principles.

First, it extends the focus from access verification to action verification. In Agentic AI environments, the key risk factor is not only whether access is granted, but also what actions an agent performs with the privileges it has been granted. Therefore, AI-ZTMM includes the execution actions of agents themselves as objects of continuous verification [30].

Second, it extends the principle of least privilege to the principle of least agency. AI-ZTMM evaluates not only whether an agent is granted the minimum privileges necessary, but also whether the scope of autonomous actions is minimized, including tool invocation scope, execution frequency, communication targets, and execution conditions [2].

Third, it reflects a multi-layer enforcement structure. Because agent actions involve input interpretation, reasoning, tool invocation, external communication, and delegation, AI-ZTMM evaluates controls at Input, Inline, Gateway, and Delegation enforcement points.

### 4.3. Evaluation Structure and Requirement Integration

#### 4.3.1. Threat Classification

This paper does not define a new threat taxonomy independently; instead, it integrates and organizes existing public threat taxonomies. Specifically, the prioritized risk categories of the OWASP Top 10 for Agentic Applications (ASI-01–ASI-10) are used as the primary analytical framework [1]. These categories are aligned with the 17 threats (T1–T17) defined in the OWASP Agentic AI Threats and Mitigations framework [22] and the five risk categories presented in the CISA multi-agency guidance [13]. Table 3 presents the correspondence among the three taxonomies. The mapping between OWASP ASI and T&M follows the official mapping provided by OWASP, whereas the mapping to the CISA risk categories was developed in this paper as a supplementary analytical step. The resulting integrated taxonomy serves as the basis for classifying and deriving security requirements.

Table 3 sets out the correspondence between the threat areas and the public taxonomies.

#### 4.3.2. Derivation of Security Requirements

From the threat areas in Section 4.3.1, this paper derives security requirements as implementation-independent control objectives. This step translates threat categories into assessable security capabilities. A requirement is therefore not a vendor-specific function or concrete implementation mechanism but a control objective that an organization should satisfy to manage one or more Agentic AI threat areas.

Each requirement is grounded in public threat taxonomies, mitigation guidance, and academic literature, and is described as an independent control objective. The unit of a requirement is defined as a recurring control perspective identified across the reviewed sources, such as agent identity management, delegation control, tool execution control, memory integrity, or action auditing. When different sources present semantically identical or similar controls, they are consolidated into a single requirement to avoid duplication. Requirements that merely repeat general IT security controls without Agentic AI-specific changes are excluded, whereas controls that require new or modified treatment due to agent autonomy, tool invocation, memory persistence, external entity interaction, or multi-agent delegation are retained.

The resulting requirements are not mapped one-to-one to individual threats. A single requirement may address multiple threat areas, and the same threat may require several requirements across different control domains. This reflects the multi-layered nature of Agentic AI security, where a single attack path may involve identity, runtime environment, network communication, application action, and data context at the same time. Through this procedure, a total of 31 requirements were derived.

Table 4 summarizes the identifiers, requirement names, supporting sources, and related threats of the 31 requirements. The related threats refer to the threat areas (ASI-01–ASI-10) in Table 3, and the supporting sources indicate the literature that supports each control objective. For brevity, detailed descriptions of the control objectives for each requirement are provided in Appendix A.

#### 4.3.3. Evaluation Structure

The evaluation structure of AI-ZTMM consists of five Pillars, embedded Cross-cutting Capabilities, and four maturity levels. It retains the CISA ZTMM five-Pillar structure while redefining the assessment targets for Agentic AI. Specifically, Devices is extended to Devices and Runtime Environments to cover agent execution foundations such as containers and sandboxes, and Applications and Workloads is extended to Applications, Workloads and Agent Actions to reflect the shift from application access control to agent action control. The remaining Pillars retain their CISA names but extend their assessment targets to Agentic AI components. General controls that apply without substantive change follow the CISA ZTMM, and Table 5 summarizes only the Agentic AI-specific redefinitions.

AI-ZTMM adopts the four maturity levels of the CISA ZTMM: Traditional, Initial, Advanced, and Optimal. These levels represent a progression from reliance on existing controls to agent-specific control, dynamic policy-based enforcement, and lifecycle-wide continuous improvement. Table 6 summarizes each level and its interpretation for Agentic AI security assessment.

In practice, an organization first identifies the agents it operates and the actions they can perform, and then assesses the Functions within each Pillar using the maturity criteria provided in Appendix B. Because the maturity levels are not aggregated into a single score, the assessment produces a Pillar-level maturity profile. Differences in maturity across Pillars and the presence of uncontrolled high-risk actions indicate areas requiring remediation. The specific procedures for scoping, evidence collection, and prioritization are left to the organization, while AI-ZTMM provides the criteria needed to support these judgments. Recurring qualifiers in the criteria, such as *real time*, are therefore not bound to fixed thresholds, since the latency that qualifies as real time differs by orders of magnitude between an OT control loop and an IT workflow. Organizations instantiate such qualifiers for their own environment during scoping.

### 4.4. Action Space and Action Risk Factors

#### 4.4.1. Action Space

Action Space refers to the range of actions that an agent can select or execute within a given operating environment. Because agents can dynamically plan tasks and combine tools at runtime, exhaustively enumerating all possible actions in advance may be difficult or costly, and static policies alone may not adequately capture them [21]. AI-ZTMM therefore does not assume that every possible action can be identified in advance. Instead, it requires organizations to classify the actions they have identified into the following three policy categories:

- Permitted actions: Low-risk actions that can be performed without prior approval
- Conditional actions: Actions that can be performed only under specific conditions or approval
- Prohibited actions: Actions that an agent must not perform

Here, high-risk actions do not constitute a separate category parallel to the three categories above. Rather, they are a risk classification identified by applying the Action Risk Factors in Section 4.4.2. In other words, when an action is classified as high-risk, it is assigned either to the conditional category, such as human approval or prior authorization, or to the prohibited category.

#### 4.4.2. Action Risk Factors

Action Risk Factors are qualitative criteria for assessing the risks of actions within the Action Space. They consider the characteristics of the action, the privileges involved, the level of autonomy, and the operational context. The framework comprises seven factors: reversibility, real-time impact, data sensitivity, external communication, privilege level, autonomy level, and impact scope. For example, the same deletion action may pose different risks depending on whether it requires human approval or is executed autonomously.

These factors are derived from the security requirements established in Section 4.3.2. Reversibility, real-time impact, data sensitivity, external communication, privilege level, and autonomy level are based on REQ-17. Impact scope was added to capture the scale of the entities, data, or systems affected by an action. Actions with otherwise similar characteristics may differ substantially in potential harm when one affects a single record and another affects an entire dataset. Each factor is rated as High, Moderate, or Low according to its contribution to action risk. A High rating for reversibility therefore means that the action is difficult to reverse or recover from, not that it is highly reversible. Table 7 presents the definition, evaluation question, and rating criteria for each factor.

AI-ZTMM does not assign fixed weights or calculate an aggregate score because factor importance varies across organizations and aggregation may obscure high-risk combinations. High-risk actions are therefore identified using the co-occurring factor patterns defined in Section 4.4.3.

#### 4.4.3. High-Risk Action Determination

High-risk actions are identified using the factor ratings assigned in Section 4.4.2 and critical-risk conditions defined by the organization in advance. As a baseline, an action is classified as high-risk when two or more of the seven factors are rated High. This threshold is not a quantitative risk value derived from the literature but an operational rule intended to support consistent identification of compound risk. The rule was refined through the expert review sessions described in Section 3.4.

Two exceptions apply. First, an action is classified as Prohibited regardless of its factor ratings if it is prohibited by law, regulation, physical safety requirements, or organizational policy, or if it does not satisfy a mandatory authorization requirement. Second, an action with only one High-rated factor may still be classified as high-risk if it meets a predefined critical-risk condition. Thus, the two-High threshold serves as a baseline and does not override explicit prohibitions or organization-specific escalation conditions.

Formally, the Action Risk Factors for an action (a) are represented by the following vector:

(1) $$F\left(a\right)=\left(R_{a},T_{a},D_{a},E_{a},P_{a},A_{a},S_{a}\right),\;F_{i}\left(a\right)\in \left\{L,M,H\right\},$$

where *R*, *T*, *D*, *E*, *P*, *A*, and *S* denote reversibility, real-time impact, data sensitivity, external communication, privilege level, autonomy level, and impact scope, each rated by its contribution to risk as defined in Section 4.4.2. Let $n_{H}\left(a\right)=\left|\left\{i\in \left\{1,\ldots ,7\right\}|F_{i}\left(a\right)=H\right\}\right|$ denote the number of factors rated High. An action is treated as high-risk when

(2) $$\mathrm{HighRisk}\left(a\right)\Leftrightarrow n_{H}\left(a\right)\geq 2\;\vee \;\mathrm{Esc}\left(a\right),$$

and its Action Space category is determined by

(3) $$C\left(a\right)=\left\{\begin{array}{cc} \mathrm{Prohibited}, & G\left(a\right)\vee (\mathrm{HighRisk}(a)\wedge \neg \mathrm{Mit}(a))\\ \mathrm{Conditional}, & \neg G(a)\wedge \mathrm{HighRisk}(a)\wedge \mathrm{Mit}(a)\\ \mathrm{Permitted}, & \mathrm{otherwise}. \end{array}\right.$$

G(a) indicates that an action is explicitly prohibited by law, regulation, physical safety requirements, or organizational policy, or that it fails to satisfy a mandatory authorization requirement. Esc(a) indicates that an action with only one factor rated High meets a predefined critical-risk condition and is therefore escalated to high-risk. Mit(a)=1 indicates that the controls required for conditional execution can reduce the residual risk to a level acceptable to the organization. It represents whether such mitigation is feasible, not whether the controls have already been applied. Mitigability is assessed based on the applicability of controls corresponding to the factors that triggered the high-risk determination, the expected residual impact after those controls are applied, and the organization’s risk acceptance criteria.

When multiple conditions apply, the Action Space categories take precedence in the following order: Prohibited, Conditional, and Permitted. High-risk status is determined by $n_{H}\left(a\right)$ and Esc(a) rather than by a weighted sum or average of the factor ratings. The final Action Space category is then determined by applying the explicit-prohibition condition G(a) and the mitigability condition Mit(a). Accordingly, the co-occurrences below illustrate common high-risk patterns rather than an exhaustive set of determination conditions.

Representative co-occurrences include the following:

- An irreversible action combined with administrative privilege, such as account deletion or modification of a privilege policy
- Sensitive data combined with transmission to an uncontrolled external destination, such as sending customer records outside the organization
- Autonomous execution combined with an immediate external effect, such as automated deployment or payment approval
- Administrative privilege combined with an unrecoverable system change, such as executing shell commands derived from retrieved documents
- Broad impact scope combined with external communication or autonomous execution, such as mass email distribution
- An irreversible action combined with an immediate physical effect, such as issuing actuator commands or transmitting industrial control commands

An action classified as high-risk is assigned at least to the Conditional category. Controls corresponding to the factors that triggered the determination are then applied, including human-in-the-loop review, prior authorization, enhanced validation of inputs, intent, and parameters, execution-environment isolation, and policy-based blocking. If these controls cannot reduce the residual risk to an acceptable level, or if the action is prohibited by law, safety requirements, or organizational policy, the action is assigned to the Prohibited category. This principle is reflected in the Pillar-specific criteria in Section 4.5.

### 4.5. Pillar-Specific Evaluation Criteria

This section presents the Functions defined for the five Pillars. Each Function is classified as Extended or New according to its relationship with the CISA ZTMM. Extended Functions adapt existing CISA control areas to Agentic AI components, such as agents, tools, memory, RAG systems, and external integrations. New Functions represent Agentic AI-specific control areas that are not addressed in the CISA ZTMM and are introduced to manage risks arising from autonomous action execution, dynamic delegation, memory manipulation, tool invocation, and interaction with external systems. Controls that apply without substantive modification follow the CISA ZTMM and are not listed separately.

Visibility and Analytics, Automation and Orchestration, and Governance are embedded within each Pillar as Cross-cutting Capabilities. Because their control objectives are consistent across Pillars, the fifteen cross-cutting Functions are consolidated in Section 4.5.6.

Each table presents the Function ID, name, type, description, and related security requirements. Maturity is assessed based on the level of control capability within each Pillar rather than on individual threats. Detailed maturity criteria are provided in Appendix B.

#### 4.5.1. Identity

The Identity Pillar governs the identities of subjects that access resources and perform actions. In Agentic AI environments, agents must be managed as independent non-human identities (NHIs) rather than as extensions of human or service accounts. This Pillar therefore covers agent identity, credential lifecycle management, and delegation control, including verification that delegated privileges do not exceed those of the delegating principal. The Functions evaluated under this Pillar are presented in Table 8.

#### 4.5.2. Devices and Runtime Environments

The Devices and Runtime Environments Pillar governs the physical and virtual execution environments on which agents operate. It extends conventional device controls to runtime environments such as containers, virtual machines, and serverless platforms, including those deployed on mobile, edge, IoT, robotic, and OT/ICS systems. The Pillar evaluates registration, isolation, integrity, and threat detection, as a compromise at any layer can undermine the trustworthiness of agent actions. The Functions evaluated under this Pillar are presented in Table 9.

#### 4.5.3. Networks

The Networks Pillar governs communication paths and network traffic. In Agentic AI environments, agents dynamically interact with external APIs, MCP servers, and other agents, making trust boundaries fluid and counterpart trust difficult to establish in advance. This Pillar therefore extends existing network controls to include integrity verification for agent communication protocols, such as MCP and A2A, and egress control at external integration points. General controls such as TLS, mTLS, and traffic resilience follow the CISA ZTMM and are not redefined. The Functions evaluated under this Pillar are presented in Table 10.

#### 4.5.4. Applications, Workloads and Agent Actions

The Applications, Workloads and Agent Actions Pillar governs the actions agents perform through applications, tools, APIs, and code execution environments. Unlike conventional application security, which is primarily access-centric, this Pillar focuses on post-access behavior and extends evaluation from access control to action-level verification.

This Pillar covers tool access control, application protection, AI software supply chain integrity, security testing, Action Space definition, intent verification, risk-based action control, and AI-generated code execution. The supply chain scope includes the provenance and integrity of models, tools, MCP descriptors, plugins, and workflows used by agents. In contrast, the Devices and Runtime Environments Pillar addresses execution environment integrity and isolation. For AI-generated code, this Pillar governs execution approval and policy enforcement, while the Devices and Runtime Environments Pillar governs the secure runtime in which the code is executed. The Functions evaluated under this Pillar are presented in Table 11.

#### 4.5.5. Data

The Data Pillar governs the protection of data used by agents. In Agentic AI environments, memory, RAG repositories, and contextual data directly influence agent decisions and actions. This Pillar therefore extends existing data controls to include memory and context integrity, protection against persistent poisoning, and end-to-end information-flow tracking. General controls such as data classification, encryption, and availability remain governed by the CISA ZTMM. The Functions evaluated under this Pillar are presented in Table 12.

#### 4.5.6. Cross-Cutting Capabilities

The three Cross-cutting Capabilities apply across all five Pillars. Rather than serving as independent assessment axes, they are embedded within each Pillar and mature according to its control level.

Visibility and Analytics monitors agent actions and data flows, Automation and Orchestration triggers responses such as blocking or isolation, and Governance manages deployment, autonomy, lifecycle, and HITL policies. Because these capabilities share the same objectives across Pillars but apply to different targets, their Functions are consolidated in Table 13 rather than repeated in Section 4.5.1, Section 4.5.2, Section 4.5.3, Section 4.5.4 and Section 4.5.5. Together with the Pillar-specific Functions, they contribute to the 41 Functions summarized in Section 5.1.

### 4.6. Application to Sensor-Driven Cyber-Physical Agentic Systems

Agentic AI is expanding beyond software workflows into hardware design and cyber-physical control. Hardware design agents generate artifacts that are realized in physical form, as in the automatic sizing of analog and mixed-signal circuits [36] and digital chip design [37], while sensor-driven control agents interpret environmental state and issue commands to actuators or control systems. In both types the agent’s output can lead to a defective device or an unsafe physical state, and AI-ZTMM evaluates both at the software action layer: for a hardware design agent, the commit of a design artifact is itself the controlled action.

These risks have already been demonstrated: an LLM agent has been used to design stealthy analog Trojans that evade conventional detection [38], poisoned training data has been shown to induce hardware description code containing backdoors [39], and work on cyber-physical AI agents identifies the agent–environment interface as a principal attack surface [40]. The threat areas of Section 4.3.1 are therefore not confined to information systems.

This section develops the sensor-driven case, whose risk path forms a four-stage chain: sensor telemetry is acquired, loaded into the agent’s context or memory, interpreted so that an action is selected, and issued as a control command that changes a physical state. The entry point and the terminal effect differ from those of a conventional software agent—the untrusted input is a physical measurement, and the terminal effect is a physical state change—so the correspondence with the existing Functions is stated explicitly in Table 14. The assessment is confined to the security controls over this chain; the behavior of the physical system once a command has been issued is treated at the end of this section.

The following example applies the Action Risk Factors of Section 4.4.2 to a sensor-driven action. A predictive-maintenance agent in a process plant collects vibration and temperature telemetry, retrieves internal maintenance procedures, and changes the setpoints of a single unit over the control network. The agent is deployed inside the OT control zone, and an interruption of coolant supply beyond a threshold duration causes irreversible thermal damage. Table 15 rates the command that closes the coolant valve; a rating denotes the level of risk arising from the factor rather than the magnitude of the property, and Privilege Level is judged on the scope of the digital privilege granted to the agent.

Reversibility, real-time impact, autonomy level, and impact scope are rated High, so $n_{H}\left(a\right)=4$. The command is not disallowed outright by law or by a safety requirement, so G(a)=0, and its residual risk can be reduced by prior human approval (AW-7), verification of consistency with the task objective and context (AW-6), and command validation at a PEP (AW-5), so that Mit(a)=1. Applying the rule of Section 4.4.3 therefore gives

(4) C(a)=Conditional.

The action matches the sixth representative pattern of Section 4.4.3, an unrecoverable action combined with immediate physical effect, and the factors rated Moderate or Low neither reduce $n_{H}\left(a\right)$ nor offset those rated High.

The data-layer Functions matter as well: had the maintenance procedure been poisoned or the telemetry spoofed, the agent would have selected the same action through reasoning that appears normal, which is why trust-boundary isolation of retrieved content (DA-5), provenance attribution (DA-4), and behavioral baselines for command patterns (AW-9) are required.

As defined in Section 1, this section evaluates only the software action layer. An action that violates a physical safety requirement is prohibited irrespective of its factor ratings under the first qualification of Section 4.4.3, and its safety consequence is reflected in the Impact Scope rating; the derivation and verification of those requirements, together with actuator control stability, real-time scheduling, sensor calibration, and functional safety assurance, lie outside AI-ZTMM.

## 5. Analytical Model Evaluation

This section reviews AI-ZTMM along two axes. The derivation traceability review in Section 5.1 examines the internal consistency of the model by tracing how the evaluation criteria are derived from threats and requirements. The scenario coverage review in Section 5.2 uses external taxonomies and public incident cases not used in model construction to examine the extent to which AI-ZTMM can account for observed attack behaviors.

### 5.1. Derivation Traceability

**Figure 2:**
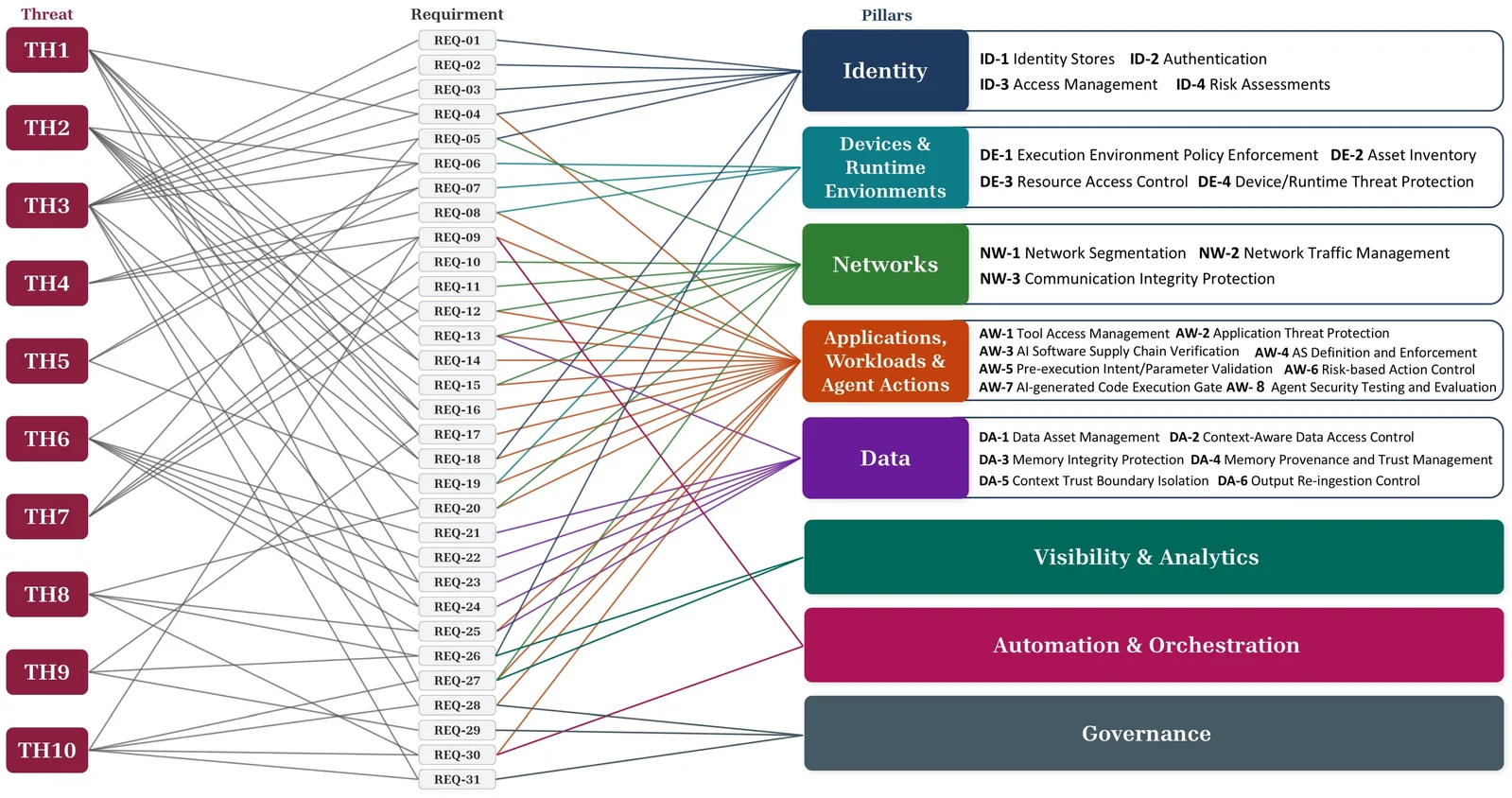
Threat–requirement–Pillar derivation traceability. The left side shows the derivation from threats (ASI1–ASI10) to requirements (REQ-01–REQ-31), and the right side shows how the requirements are assigned to the Pillars and Cross-cutting Capabilities.

AI-ZTMM follows a one-way derivation structure that runs from threats (ASI1–ASI10) to security requirements (REQ-01–REQ-31), to 41 security Functions, and finally to the five Pillars and three Cross-cutting Capabilities. This section analyses the consistency of the derivation chain, the distribution of new Functions, the coverage of each threat, and the structure of the cross-cutting requirements. The overall derivation path is shown in Figure 2.

Derivation consistency. All 10 threats and all 31 requirements are linked to at least one Function, and all 41 Functions can be traced back to a requirement and a threat. There are therefore no unlinked or orphaned elements, and the derivation path contains no backward dependency or circular reference.

Distribution of new Functions. As shown in Table 16, 32 of the 41 Functions are extensions grounded in the CISA ZTMM and 9 were newly introduced for Agentic AI. Eight of the nine new Functions (88.9%) are concentrated in Applications, Workloads and Agent Actions and in Data. This indicates that the new control obligations of Agentic AI arise mainly from the execution of agent actions and from the handling of data and memory, whereas Identity, Devices and Runtime Environments, and Networks are addressed principally by extending existing controls. The distribution therefore supports, at the level of control structure, the paper’s claim that Zero Trust control should extend from access to action.

Threat coverage. All 10 threats are addressed by Functions spanning two or more Pillars, and no threat is isolated within a single Pillar. ASI3 and ASI10 are addressed across all five Pillars through 15 and 12 Functions respectively, whereas ASI5 and ASI7 are concentrated in two Pillars. Control is thus distributed in proportion to how widely a threat propagates, which reflects a defense-in-depth structure in the model.

Cross-cutting requirements. REQ-09 and REQ-26 are implemented across five Pillars and REQ-31 across four, and each corresponds to one of the three Cross-cutting Capabilities. This shows that the design of Section 4.5.6, which embeds auditability, automated response, and governance within each Pillar rather than separating them as independent assessment axes, is also reflected in the actual placement of the Functions.

### 5.2. External Scenario-Based Coverage Assessment

This section examines whether AI-ZTMM covers attack stages documented in public cases involving LLMs, generative AI, and agents. The analysis focuses on content coverage and does not test whether maturity levels predict security outcomes. MITRE ATLAS, a public knowledge base of adversarial tactics and techniques for AI systems, is used as an external reference because it was not used in model development [41]. Its relevance is also supported by CISA guidance and a DHS report on adversarial AI [42,43], reducing the risk of circular evaluation.

The analysis proceeds in two steps. First, coverage is assessed across the ATLAS case base. Second, three cases, selected purposively rather than as a statistical sample, are examined in depth: Amazon Q Developer Extension (AML.CS0047), postmark-mcp (AML.CS0053), and SesameOp (AML.CS0042). These cases represent supply chain compromise leading to destructive actions, data exfiltration through a malicious tool and MCP server, and command-and-control abuse of an external AI service. The cases are decomposed into finer-grained stages for qualitative analysis, but these stages are excluded from the quantitative coverage calculation.

#### 5.2.1. Coverage Across the ATLAS Case Base

The analysis uses the 63 public Case Studies(AML.CS0000–AML.CS0062) available on the MITRE ATLAS website as of 30 June 2026. Cases were included when an LLM, generative model, or autonomous agent was the attacked or abused component. Cases involving classical machine-learning classifiers, computer-vision systems, or biometric systems were excluded. Supply chain cases were retained only when the compromised component was connected to an agent runtime, tool, plugin, or MCP component; cases limited to general machine-learning infrastructure or model repositories were excluded because the AI-ZTMM supply chain Functions apply to agent-side components. Applying these criteria yielded 38 cases comprising 326 documented attack stages. Although not all retained cases involve Agentic AI exclusively, all fall within the control scope of AI-ZTMM.

Before assessing coverage, the stages were divided into three bands according to predefined rules based on their ATLAS tactics. Band A includes attacker-side preparation categorized as Reconnaissance, Resource Development, or AI Attack Staging. Band B includes Impact stages that describe a resulting state rather than an independent action. Band C includes attack execution and follow-on activity and is the set assessed for relevance to organization-side controls. Table 17 summarizes the distribution.

Each Band C stage was assessed against the Functions of AI-ZTMM and, for comparison, the CISA ZTMM. A stage was classified as *addressed* when at least one Function directly targeted the exploited mechanism, and as *partially addressed* when a Function restricted a stage-specific precondition or channel without directly targeting the mechanism. Generic controls applicable without modification to unrelated systems or attacks did not qualify as coverage. A stage was classified as *not addressed* when neither the mechanism nor a stage-specific precondition or channel was targeted.

Two coders independently applied the predefined codebook to all 211 Band C stages (File S2). For each model, they recorded the coverage level, relevant AI-ZTMM Functions, and rationale for the judgment. Inter-coder agreement was measured using simple percentage agreement and linearly weighted Cohen’s κ, with not addressed (X), partially addressed (T), and addressed (O) treated as ordinal categories. Before adjudication, agreement for AI-ZTMM was 84.4% with κ=0.660, while agreement for the CISA ZTMM was 89.6% with κ=0.852. Under the interpretation scale of [44], these values indicate substantial and almost perfect agreement, respectively. Disagreements were resolved through discussion of the recorded rationales and Function mappings, and the adjudicated results were used in the final analysis.

Coverage is instead reported in three steps: the stages left unaddressed by the CISA ZTMM, the proportion of this residual set addressed by AI-ZTMM, and the remaining gap after combining both models. Table 18 presents the results.

In the Combined assessment, a stage was classified as *addressed* if either model addressed it, *partially addressed* if neither model addressed it but at least one partially addressed it, and *not addressed* only if both models left it unaddressed.

The CISA baseline did not address 126 of the 211 Band C stages, which were concentrated in the prompt and agent-action layers. The most frequent techniques were direct or indirect prompt injection (25 stages), agent tool invocation (15), prompt infiltration through public-facing applications (11), prompt obfuscation (9), and access to a public AI product or service (8). These counts represent stage occurrences and include only the most frequent techniques; therefore, they do not sum to 126. AI-ZTMM addressed 95 of these stages (75.4%) and partially addressed another 5 (4.0%). This residual coverage is the principal indicator of the analysis.

Across all 211 Band C stages, AI-ZTMM addressed 136, partially addressed 9, and did not address 66. Of the 66 stages it did not address, the CISA baseline addressed or partially addressed 40. These stages mainly involved conventional concerns, such as valid-account abuse, general credential access, and endpoint compromise, which AI-ZTMM leaves to the CISA baseline by design. The remaining 26 stages were addressed by neither model.

These 26 stages included normal interaction with public AI products or inference APIs (11), discovery through repeated observation of model responses (6), general environment enumeration (2), training-data or data-supply-chain poisoning (2), and five additional stages involving model extraction, citation manipulation, or activity on AI services outside the assessed organization’s control. These stages either use legitimate interfaces, belong to the model development pipeline, fall outside the organization’s control boundary, or are not directly targeted by either model.

Taken together, these results support two conclusions. First, the gap left by the CISA baseline is empirically located in the prompt and agent-action layers, which account for 59.7% of the organization-side stages in the retained cases. Second, the Functions defined in Section 4.5 address 75.4% of this gap, indicating that AI-ZTMM responds to attack behaviors documented in public cases rather than to a purely hypothetical threat model. Because MITRE ATLAS was not used in model development, this assessment avoids circularity. However, the analysis demonstrates content coverage rather than control effectiveness: an addressed stage indicates that a relevant evaluation criterion exists, not that the control has been implemented or would prevent the attack.

#### 5.2.2. Case 1: Amazon Q Developer Extension Supply Chain Compromise

In July 2025, malicious code was inserted into the open-source repository of Amazon Q Developer Extension for VS Code, AWS’s AI coding assistant, and was included in the official release version v1.84.0 (CVE-2025-8217). The attacker used an inappropriately scoped GitHub token to merge an untrusted pull request. The merged code contained a natural-language prompt instructing the embedded AI agent to act as a “system cleaner” and delete local files and cloud resources, including S3 buckets, EC2 instances, and IAM users. Although the extension had been installed approximately 960,000 times, syntax error prevented actual destructive actions. This incident shows how a compromised supply chain component can redirect an agent toward destructive actions. Table 19 maps this incident (AML.CS0047) to ATLAS techniques and AI-ZTMM evaluation components.

The mapping shows that the initial credential abuse is handled by the CISA baseline, while the stages from the supply chain entry onward require AI-ZTMM controls across Devices and Runtime Environments, Applications and Workloads, Data, and the embedded visibility, automation, and governance slots. In particular, the destructive action stage is a typical high-risk action in which multiple Action Risk Factors are combined: reversibility, due to irreversible deletion; impact scope, due to broad resource impact; and privilege level, due to cloud administrative authority. This shows that the qualitative high-risk determination principle in Section 4.4.3 captures the incident’s risk structure. In addition, the incident chain of “supply chain entry → action redirection through prompt injection → autonomous execution → irreversible impact” supports the rationale for AI-ZTMM’s Multi-layer Enforcement design, which attempts to block attacks at multiple points, including supply chain verification, intent verification, autonomy control, and differentiated action-risk control.

#### 5.2.3. Case 2: Data Exfiltration Through the Postmark-Mcp Malicious MCP Server

In September 2025, postmark-mcp, an MCP server package that enables AI assistants to send emails, was maliciously modified and distributed through npm. The attacker copied the legitimate MCP server, published an impersonated package under the same name, and maintained normal operation across 15 versions to accumulate trust. In version 1.0.16, the attacker added a single line of code that secretly BCCed all outgoing emails to an external address, leaking sensitive communications such as password reset links, MFA codes, and invoices. Because the package used legitimate sending infrastructure and differed by only one line, the attack bypassed traditional email authentication controls and was difficult to detect through static or dependency scanning. This incident was reported as the first publicly documented real-world malicious MCP server case. Table 20 maps this incident (AML.CS0053) to ATLAS techniques and AI-ZTMM evaluation components.

The mapping shows that the attack stages of this incident can be explained across Applications and Workloads, Data, Networks, Identity, and the visibility and governance slots. This case particularly supports the validity of two evaluation criteria. First, it clearly shows that risk is amplified by the combination of Action Risk Factors: external transmission through email BCC and data sensitivity involving authentication codes and financial information. This indicates that the Tool-level action decomposition in Section 4.4.2 can capture real exfiltration paths. Second, the fact that both traditional email authentication and static scanning were ineffective shows that static controls such as signatures and authentication alone cannot detect malicious activity routed through legitimate infrastructure. Behavior-based anomaly detection through Visibility and external communication control through Networks are required together. This supports the structure of AI-ZTMM, which evaluates static integrity verification and dynamic behavioral monitoring through distinct but complementary Pillars.

#### 5.2.4. Case 3: SesameOp Command-and-Control Abuse of an External AI Service

In 2025, Microsoft Incident Response (DART) discovered SesameOp, a backdoor that abused the OpenAI Assistants API as a covert command-and-control (C2) channel on compromised systems. The attacker maintained persistence for several months through a web shell and a compromised Visual Studio utility. SesameOp received and executed commands through the OpenAI Assistants API, exfiltrated encrypted results through the same channel, and deleted the Assistants and Messages used for communication to remove traces. Unlike the previous two cases, the initial access and persistence stages of this attack were based on traditional system compromise. Therefore, the entire attack chain cannot be explained by this model.Although AML.T0096 is not directly addressed by AI-ZTMM under the quantitative coding criteria, analysis of communication paths identifies AI-ZTMM controls that may support defense-in-depth at specific points.

Table 21 presents the decomposition of this incident (AML.CS0042) into ATLAS techniques and its mapping to the evaluation components of AI-ZTMM.

The mapping shows that the initial access and persistence stages of this incident are outside the assessment scope of the model, but AI-ZTMM controls can intervene at the stage where C2 traffic passes through an external AI service, namely the Assistants API. However, destination allowlists alone are insufficient to block such communication because the traffic is directed to a legitimate AI service endpoint and is encrypted. Blocking or detecting this communication pattern requires identity-bound egress control (NW-2) and behavior-based anomaly detection (NW-4, AW-9). These controls distinguish authorized AI-service use from abnormal AI-API communication. These controls correspond to the higher maturity levels of AI-ZTMM Networks and Visibility. This case therefore shows that organizations equipped with such controls may detect or block attacks that abuse external AI services at the C2 stage. At the same time, because the initial system compromise is not covered by this model, the case also clarifies the boundary of AI-ZTMM’s applicability.

## 6. Discussion

### 6.1. Limitations

This paper has four main limitations. First, AI-ZTMM was developed through a literature-based design and analytical approach. Although expert review, derivation traceability, and scenario-based coverage assessment were used to examine its consistency and applicability, the model has not been validated through implementation or organizational deployment. Second, AI-ZTMM covers only the software action layer of agents deployed in physical systems. It does not address physical-layer properties such as actuator stability, real-time constraints, sensor reliability, and functional safety. Third, AI-ZTMM is a qualitative self-assessment model rather than a quantitative scoring or certification framework. It identifies control gaps and improvement priorities through Pillar-level maturity profiles but does not provide quantitative benchmarking or formal compliance assessment.

### 6.2. Future Work

Future work should proceed in three directions. First, AI-ZTMM should be applied to real organizational environments and concrete agent deployment cases to validate the practicality of the proposed Functions, maturity levels, and self-assessment procedure. Second, assessment support tools can be developed to improve the repeatability and usability of the model. These tools may support agent inventory construction, Action Space identification, Action Risk Factor evaluation, and Pillar-level maturity profiling. Third, the model should be extended to Physical AI environments. This requires adding separate Pillars or criteria for actuator trust, real-time control stability, sensor reliability, functional safety, and operational continuity. This extension would integrate software action control with physical and communication-layer security in one maturity framework.

## 7. Conclusions

This paper proposed the Agentic AI Zero Trust Maturity Model (AI-ZTMM), which inherits the five-Pillar structure of the CISA Zero Trust Maturity Model while integrating the risks of autonomous agent actions into maturity assessment. The proposed model extends Zero Trust trust assessment from access-centric verification to action-centric verification and introduces Action Space and Action Risk Factors as evaluation axes for assessing the scope and risk characteristics of agent actions. This paper presented a one-way derivation chain from threats to requirements, security Functions, Pillars, and Cross-cutting Capabilities. It defined an evaluation structure with 10 threats, 31 requirements, and 41 security Functions and operationalized each Function into four maturity levels.

The analytical evaluation showed that the proposed evaluation criteria are consistently derived from the identified threats and requirements through derivation traceability. The scenario coverage analysis further showed that attack stages in real-world Agentic AI incidents can be explained through multiple Pillars and Action Risk Factors. These results suggest that AI-ZTMM can help organizations adopt Agentic AI while maintaining continuity with existing Zero Trust maturity assessment, self-assessing autonomous action-oriented systems, and identifying improvement priorities. What these results do not establish is control effectiveness: none of them demonstrates that an organization assessed at a higher maturity level in fact experiences fewer or less severe incidents, which would require longitudinal observation of organizations operating agents under the model. Future empirical studies and extensions to Physical AI environments can further refine the applicability and scope of the proposed model.

## Figures and Tables

**Table 1:** Comparison of existing approaches and this paper (AI-ZTMM).

Approach	Continuity with CISA Five Pillars	Agent Action-Level Assessment	Organizational Self-Assessment	Maturity Levels
CISA ZTMM [5]	◯	×	◯	◯
Studies on individual controls and techniques [6,17,18,20,25]	×	∆	×	×
Agentic ZT architecture proposals [8,24,26,27,28,29,30]	×	∆	×	×
Agent trust-stage frameworks [2,14]	×	◯	∆	◯
Studies on Agentic AI risk assessment [31]	×	◯	∆	×
This Paper (AI-ZTMM)	◯	◯	◯	◯

◯: explicitly satisfied, ∆: partially satisfied, ×: not addressed

**Table 2:** Composition of the expert panel.

Expert	Position	Affiliation Type
1	Associate Professor	Academia
2	Associate Professor	Academia
3	Assistant Professor	Academia
4	Principal Researcher	Government-funded Research Institute
5	Principal Researcher	Government-funded Research Institute
6	Chief Executive Officer	Private Sector (AI Security Solutions)
7	Executive Director	Quasi-governmental Agency
8	Executive Director	Quasi-governmental Agency
9	Team Lead	Quasi-governmental Agency
10	Senior Researcher	Quasi-governmental Agency
11	Researcher	Quasi-governmental Agency

**Table 3:** Correspondence between Agentic AI threat areas and public threat taxonomies. The ASI–T&M mapping follows the official OWASP mapping, while the linkage between CISA risk categories and the threat areas is an auxiliary analytical mapping developed in this paper.

Threat Area	OWASP ASI [1]	OWASP T&M [22]	CISA Risk Category [13]
Agent Goal Hijack	ASI-1	T6, T7	Behavioral
Tool misuse & Exploitation	ASI-2	T2, T4, T16	Design & Configuration/Behavioral
Identity & Privilege Abuse	ASI-3	T3	Privilege
Agentic Supply Chain Vulnerabilities	ASI-4	T17, T2, T11, T12, T13, T16	Structural
Unexpected Code Execution	ASI-5	T11	Behavioral
Memory & Context Poisoning	ASI-6	T1, T4, T6, T12	Structural/Behavioral
Insecure Inter-Agent Communication	ASI-7	T12, T16	Structural
Cascading Failures	ASI-8	T5, T8	Structural
Human-Agent Trust Exploitation	ASI-9	T7, T8, T10	Behavioral/Accountability
Rogue Agent	ASI-10	T13, T14, T15	Behavioral/Accountability

**Table 4:** Summary of derived security requirements (REQ-01–REQ-31).

REQ ID	Requirement Name	Source	Threat
REQ-01	Issuance of unique agent identity	[1,2,6]	ASI-3
REQ-02	Agent credential lifecycle management	[1,6,22]	ASI-3
REQ-03	Delegation boundary verification and privilege ceiling	[2,18,22]	ASI-3
REQ-04	Action–intent binding	[1,18,22]	ASI-1, 3
REQ-05	Mutual authentication between agents	[1,22,27]	ASI-3, 7
REQ-06	Agent execution environment isolation	[1,22,29]	ASI-2, 3, 5
REQ-07	Runtime environment integrity & compliance monitoring	[2,22,29]	ASI-4, 5
REQ-08	AI software supply chain verification	[1,22,25]	ASI-4
REQ-09	Emergency invalidation and isolation response	[1,14,22]	ASI-4, 6, 10
REQ-10	Protection of agent communication integrity	[1,22,28]	ASI-7
REQ-11	Protocol version and schema verification	[1,22]	ASI-7
REQ-12	External entity trust verification	[1,10,22]	ASI-2, 7
REQ-13	Isolated processing of untrusted external data	[1,22]	ASI-1
REQ-14	Explicit definition of Action Space	[1,13,21,30]	ASI-1, 2
REQ-15	Tool-specific least-privilege profiles	[1,17,22]	ASI-2
REQ-16	Pre-execution intent verification	[1,17,22,26]	ASI-1, 2
REQ-17	Action risk-based differentiated approval	[13,22]	ASI-1, 2, 9
REQ-18	Just-in-time privilege granting	[1,22,28]	ASI-2, 3
REQ-19	AI-generated code execution control	[1,4,22]	ASI-5
REQ-20	Action rate and frequency limiting	[1,22]	ASI-2, 8
REQ-21	Validation of memory write content	[1,20,22]	ASI-6
REQ-22	Memory provenance and trust management	[1,20,22]	ASI-6
REQ-23	Context integrity and information flow tracking	[1,13,20,22]	ASI-1, 6
REQ-24	Context-aware data access control	[7,13,20,22]	ASI-3, 6
REQ-25	Prevention of reinjection of self-generated outputs	[1,22]	ASI-6, 8
REQ-26	Cryptographic audit logs	[1,13,22]	ASI-8, 9
REQ-27	Agent behavioral baseline and anomaly detection	[1,4,19,22]	ASI-1, 10
REQ-28	Autonomy level management and deployment approval	[1,2,13,14]	ASI-3, 10
REQ-29	HITL optimization and approval fatigue prevention	[1,22]	ASI-9
REQ-30	Multi-agent pipeline trust management	[1,9,14,19]	ASI-8, 10
REQ-31	Agent lifecycle governance	[2,13,14]	ASI-3, 10

**Table 5:** Agentic AI-oriented definition of AI-ZTMM Pillars and Cross-cutting Capabilities.

Pillar	Assessment Target from the Agentic AI Perspective
Identity	Agent ID, NHI credentials, delegation chain, session binding, action accountability
Devices & Runtime Environments	Agent runtime, containers, sandboxes, code execution environments, execution environment integrity
Networks	External APIs, MCP servers, inter-agent communication, external integration boundaries, egress control
Applications, Workloads & Agent Actions	Action Space, tool invocation, intent verification, parameter validation, workflow control
Data	Memory, RAG, context, data provenance, sensitive information protection, output control
Visibility & Analytics	Agent action logs, delegation tracing, communication tracing, anomalous behavior detection
Automation & Orchestration	Automated policy enforcement, trust-state update, automated isolation and response
Governance	Agent deployment approval, autonomy level management, lifecycle management, HITL policy

**Table 6:** Four qualitative maturity levels of AI-ZTMM.

Level	Meaning from the Agentic AI Perspective
Traditional	No separate control framework exists for agents, or agent controls are absorbed into existing account and workload controls. Shared service accounts are used, and no separate audit mechanism exists for agent actions.
Initial	Agents are recognized as separate management targets, and basic controls are introduced. Agent-specific identifiers are assigned, and allowlists for major tools are managed.
Advanced	Agent actions are dynamically controlled based on policy. Multiple PEPs, action risk classification, and delegation chain controls are implemented.
Optimal	Trust-based controls are applied across the entire agent lifecycle. Rogue agent detection, feedback loops, and continuous improvement are operated.

**Table 7:** Action Risk Factors and Their Associated Definitions, Evaluation Questions, and Levels of Contribution to Risk.

Risk Factor	Definition	Evaluation Question	Levels (Contribution to Risk)
Reversibility	Whether the action can be undone or causes permanent consequences	Can the outcome of the action be canceled or restored afterward?	H: cannot be undone. M: recoverable through a defined procedure. L: reversible by a routine operation.
Real-time Impact	Whether the outcome of the action immediately affects external entities or systems	How quickly does the outcome of the action change the state of an external entity or system?	H: external state changes at execution or within seconds. M: state changes after a delay, queue, or staged rollout. L: does not directly change external state.
Data Sensitivity	The sensitivity level of the data handled by the action	Does the action process or transmit sensitive or confidential data?	H: personal, credential, or regulated data. M: internal business data. L: public or non-sensitive data.
External Communication	Whether the action transmits data or commands outside the trust boundary	Are data or commands transmitted outside the organization’s trust boundary?	H: leaves the trust boundary to an uncontrolled destination. M: leaves to an allowlisted external party. L: remains inside the trust boundary.
Privilege Level	The scope of privileges required by the action	Does the action involve high-privilege or administrative operations?	H: administrative or policy-changing privilege. M: write access within a bounded scope. L: read-only.
Autonomy Level	Whether the action requires human approval or is executed autonomously	Does the agent execute the action independently without human intervention?	H: executed without human confirmation. M: executed with post-hoc notification or sampling review. L: requires explicit approval before execution.
Impact Scope	The scale of the target affected by the action	Does the action affect a single target, or does it affect the organization, customers, or critical systems as a whole?	H: organization, customers, or critical systems. M: a team, service, or dataset. L: a single record or resource.

H: High, M: Moderate, L: Low

**Table 8:** Functions of the Identity Pillar.

Function ID	Function Name	Type	Description	Related REQ
ID-1	Identity stores	Extended	Register AI agents as unique NHIs, manage their privilege scope, purpose, and owner, and block unauthorized agents.	REQ-01
ID-2	Authentication	Extended	Verify agent identities for access to resources, tools, and other agents, and enforce mutual, federated, and credential lifecycle controls.	REQ-02, REQ-05
ID-3	Access management	Extended	Grant JIT tool access, adjust privileges and token scopes by risk, and prevent delegated privileges from exceeding the delegating principal.	REQ-03, REQ-18
ID-4	Risk assessment	Extended	Assess agent risk using behavior, goal alignment, execution context, credentials, and runtime state as inputs for real-time authorization.	REQ-18
ID-5	Delegation chain control	New	Verify delegation boundaries and enforce limits on re-delegation, chain depth, and validity period to prevent privilege propagation.	REQ-03

**Table 9:** Functions of the Devices & Runtime Environments Pillar.

Function ID	Function Name	Type	Description	Related REQ
DE-1	Execution foundation policy enforcement	Extended	Verify the integrity of agent execution foundations and restrict or isolate execution on compromised or low-trust foundations.	REQ-07
DE-2	Asset inventory	Extended	Inventory agent hosts, physical devices, and runtime environments with unique identifiers, track their lifecycle and owning agents, and verify dependency provenance.	REQ-06, REQ-07
DE-3	Resource access control	Extended	Restrict resources accessible to the execution foundation, isolate agents, tools, and code execution, and reset session state to prevent privilege escalation and data leakage.	REQ-06
DE-4	Threat protection	Extended	Detect and block runtime threats such as sandbox escape, unauthorized system calls, runtime constraint bypass, and device-level compromise.	REQ-07

**Table 10:** Functions of the Networks Pillar.

Function ID	Function Name	Type	Description	Related REQ
NW-1	Network segmentation	Extended	Segment agent communication paths by business function and trust level, and control access based on agent identity and execution context.	REQ-12
NW-2	Network traffic management	Extended	Verify external communication targets, enforce egress allowlists, and limit call rates to prevent unauthorized access and resource exhaustion.	REQ-12, REQ-20
NW-3	Communication integrity protection	Extended	Sign and verify agent messages, detect protocol-level tampering and injection, and enforce approved protocol versions.	REQ-10, REQ-11

**Table 11:** Functions of the Applications, Workloads & Agent Actions Pillar.

Function ID	Function Name	Type	Description	Related REQ
AW-1	Tool access management	Extended	Manage invocable tools through allowlists and tool-specific least-privilege profiles, and block unauthorized invocations.	REQ-15
AW-2	Application threat protection	Extended	Treat external data as untrusted input and block prompt injection, instruction tampering, and jailbreaks before they affect agent actions.	REQ-13
AW-3	AI software supply chain verification	Extended	Verify the provenance and integrity of models, tools, MCP descriptors, and plugins using signatures, attestations, and AIBOMs, and reject unverified components.	REQ-08
AW-4	Agent security testing and evaluation	Extended	Test prompt injection robustness, intent drift, and tool misuse before and after deployment, and use the results to support deployment decisions.	REQ-28
AW-5	Action scope definition and enforcement	New	Classify the agent’s Action Space into Permitted, Conditional, and Prohibited actions, and block out-of-scope actions before execution.	REQ-14
AW-6	Pre-execution intent verification	New	Verify that each tool invocation is consistent with the task context and validate its arguments and parameters before execution.	REQ-16, REQ-04
AW-7	Differentiated action-risk control including autonomy	New	Classify actions by reversibility, data sensitivity, external communication, privilege, impact scope, and autonomy, and apply HITL and enhanced verification to high-risk actions	REQ-17
AW-8	AI-generated code execution gate	New	Separate code generation from execution, block unverified code, and permit execution only after approval and under restricted-runtime requirements.	REQ-19

**Table 12:** Functions of the Data Pillar.

Function ID	Function Name	Type	Description	Related REQ
DA-1	Data asset management	Extended	Inventory and classify agent-accessible data, including memory, RAG corpora, and context, and track data flows to identify unauthorized leakage paths.	REQ-23
DA-2	Context-aware data access	Extended	Control data access based on task purpose, execution context, and agent trust level, and block access unnecessary for the current task.	REQ-24
DA-3	Memory integrity protection	New	Inspect writes to long-term memory and RAG stores to block poisoned content, and isolate memory across sessions to prevent unauthorized knowledge transfer.	REQ-21
DA-4	Memory provenance and trust management	New	Record the provenance of memory updates, assign trust levels to entries, and expire or decay unverified memory to limit persistent poisoning.	REQ-22
DA-5	Context trust boundary isolation	New	Separate trusted and untrusted contextual data and require verification before untrusted data can influence agent decisions or actions.	REQ-23
DA-6	Output re-ingestion control	New	Prevent unverified agent outputs from being re-ingested into trusted memory or context to mitigate self-reinforcing poisoning and cascading hallucinations.	REQ-25

**Table 13:** Cross-cutting Functions.

Capability	Control Objective Within Each Pillar	Related REQ
Visibility & Analytics	Record and analyze the objects governed by each Pillar—identity and delegation, execution environments, external communication, agent actions, tool invocations, and data and memory access—to detect anomalies against established baselines.	REQ-26 REQ-27
Automation & Orchestration	Automatically coordinate Pillar-specific responses to anomalies or compromise, such as credential invalidation, runtime isolation, connection or action blocking, kill switches, and removal of poisoned content.	REQ-09 REQ-30
Governance	Establish and review policies for the agent-specific objects governed by each Pillar. For Applications, Workloads and Agent Actions, this also includes Action Space approval, Autonomy Level management, and HITL policy.	REQ-31 REQ-28 REQ-29

**Table 14:** Risk chain of sensor-driven agents and the corresponding Functions.

Stage	Characteristic Risk	Corresponding Functions
Sensor acquisition and delivery	Compromised or manipulated sensors, signal spoofing and jamming, tampering with telemetry	DE-1, DE-2, DE-4 verify the integrity of the device and its execution foundation; NW-1 and NW-3 segment and protect the delivery path; DA-4 manages the provenance of the measurements
Context and memory loading	Trust in manipulated telemetry and persistence of contamination across sessions	DA-3, DA-4, DA-5
Interpretation and action selection	Inappropriate control decisions arising from goal drift or misinterpretation	AW-2, AW-6
Control command issuance	Irreversible physical effect and consequences for operational safety	AW-5, AW-7, AW-10

**Table 15:** Application of the Action Risk Factors to a coolant-valve closure command.

Risk Factor	Property	Rating	Basis for the Rating
Reversibility	Outcome recovery limited	High	The valve can be reopened, but thermal damage after the threshold duration cannot be undone
Real-time Impact	Immediate	High	The process state changes as the command is issued
Data Sensitivity	Internal	Moderate	Process telemetry and maintenance records are used
External Communication	None	Low	The command remains inside the OT trust boundary
Privilege Level	Bounded write access	Moderate	Only the setpoints of one pre-approved unit are changed
Autonomy Level	Fully autonomous	High	Executed without operator confirmation
Impact Scope	Beyond the unit	High	Affects downstream units and plant safety

**Table 16:** Distribution of extended and new security Functions by Pillar.

Pillar	Extended	New	Total
Identity	7	1	8
Devices and Runtime Environments	7	0	7
Networks	6	0	6
Applications, Workloads and Agent Actions	7	4	11
Data	5	4	9
Total	32	9	41

**Table 17:** Distribution of attack stages across the 38 retained ATLAS cases.

Band	Description	Stages	Share
A	Attacker-side preparation, occurring outside the assessed organization	81	24.85%
B	Stages describing the outcome or effect of an action already performed rather than an independent attack action	34	10.43%
C	Attack execution and follow-on activity, coded for relevance to an organization-side control model	211	64.72%
	Total	326	100.00%

**Table 18:** Coverage of the 211 Band C stages, reported as baseline, extension, and combination.

Step	Basis of Assessment	Denom.	Addressed	Partially Addressed	Not Addressed
Baseline	CISA ZTMM over all Band C stages	211	78 (37.0%)	7 (3.3%)	126 (59.7%)
Extension	AI-ZTMM over the stages the CISA ZTMM does not address	126	95 (75.4%)	5 (4.0%)	26 (20.6%)
Combined	CISA ZTMM and AI-ZTMM over all Band C stages	211	178 (84.4%)	7 (3.3%)	26 (12.3%)

**Table 19:** ATLAS technique decomposition and AI-ZTMM mapping of the Amazon Q incident. Entries without AML identifiers are author-defined control points for qualitative analysis.

Attack Stage	ATLAS Technique/Tactic	CISA ZTMM	Corresponding AI-ZTMM Evaluation Components
Acquisition of an improperly scoped GitHub token	Unsecured Credentials (AML.T0055, Credential Access)	◯	Delegated to the CISA baseline: abuse of a repository credential is a conventional mechanism that Section 4.5 does not list as a separate AI-ZTMM Function
Inclusion of malicious code in the extension release	AI Supply Chain Compromise (AML.T0010)	◯	AW-3: provenance verification of AI software, including extensions and plugins DE-1: integrity of execution and build foundations
Prompt injection inducing the AI agent to perform destructive actions	LLM Prompt Injection (AML.T0051)	×	AW-2: prompt threat detection AW-6: pre-execution intent verification and intent binding DA-5: context trust boundary isolation
Autonomous execution in non-interactive full-tool-trust mode	Autonomy and HITL bypass	×	AW-11: Autonomy Level policy AW-7: differentiated action-risk control based on autonomy level
Attempted deletion of files and cloud resources	Impact tactic (resource destruction)	×	AW-7: differentiated action control based on reversibility and impact scope AW-9: action auditing AW-10: automated blocking and kill switch

CISA ZTMM coverage: ◯: addressed by existing CISA ZTMM controls ×: not addressed.

**Table 20:** ATLAS technique decomposition and AI-ZTMM mapping of the postmark-mcp incident. Entries without AML identifiers are author-defined control points for qualitative analysis.

Attack Stage	ATLAS Technique/Tactic	CISA ZTMM	Corresponding AI-ZTMM Evaluation Components
Copying the legitimate package name and publishing under the same name on npm	Impersonation (AML.T0073, Defense Evasion)	◯	AW-3: provenance verification of Tools and MCP servers ID-2: authentication of identities associated with Tool access
Adding a backdoor after trust accumulation (rug pull)	AI Supply Chain Rug Pull (AML.T0109, Defense Evasion)	◯	AW-3: integrity verification of MCP descriptors and Tool definitions, and re-verification upon version changes
External BCC exfiltration of all emails	Exfiltration via AI Agent Tool Invocation (AML.T0086, Exfiltration)	∆	DA-1: data asset and information flow management. NW-2: external communication (egress) control and blocking of unauthorized destinations
Bypassing traditional controls through legitimate infrastructure	Defense Evasion	∆	AW-9: behavior-based anomaly detection, including anomalous recipients and external transmission patterns NW-4: communication-based anomaly detection and egress monitoring
MCP server operating with broad privileges	Excessive privilege	∆	ID-3: limitation of privilege and token scope AW-1: Tool-specific least-privilege control)

CISA ZTMM coverage: ◯: addressed by existing CISA ZTMM controls, ∆: partially addressed but without an Agentic AI-specific control point.

**Table 21:** ATLAS technique decomposition and AI-ZTMM mapping of the SesameOp incident. Entries without AML identifiers are author-defined control points for qualitative analysis.

Attack Stage	ATLAS Technique/Tactic	CISA ZTMM	Corresponding AI-ZTMM Evaluation Components
Initial access and persistence through a web shell and compromised Visual Studio utility	Initial Access and Persistence tactics	◯	Out of scope: traditional endpoint and development-tool compromise, not an operational agent evaluated by this model
OpenAI Assistants API abuse for C2 commands and encrypted result exfiltration	AI Service API (AML.T0096, Command and Control)	◯	NW-2: supporting egress control for external AI-service communication
Anomalous communication patterns such as repeated Assistants API calls	C2 communication	∆	NW-4: supporting anomaly detection for abnormal external communication patterns AW-9: supporting behavioral monitoring of anomalous actions and communication patterns
Trace removal through deletion of Assistants and Messages	Defense Evasion tactic	×	Out of scope: logging and control domain of the external AI service provider

CISA ZTMM coverage: ◯: addressed by existing CISA ZTMM controls, ∆: partially addressed but without an Agentic AI-specific control point, ×: not addressed.

## Data Availability

The data analyzed in this study are publicly available through the MITRE ATLAS Case Studies at https://atlas.mitre.org/studies, accessed on 30 June 2026.

## Supplementary Materials

The following supporting information can be downloaded at https://www.mdpi.com/article/10.3390/s26165205/s1: File S1: Derivation codebook and requirement-to-Pillar coding record; File S2: ATLAS coverage coding data.

## Appendix A. Definitions of Security Requirements

This appendix provides the detailed control objective descriptions of the 31 security requirements summarized in Table 4. Each requirement was derived from the threat areas organized in Section 4.3.1 and consolidated from semantically similar controls identified in public threat taxonomies, mitigation guidance, and academic literature. The definitions in this appendix specify the intended control objective of each requirement and serve as the basis for mapping the requirements to Pillar-specific Functions and maturity criteria. Table A1 states them.

**Table A1:** Detailed definitions of derived security requirements (REQ-01–REQ-31).

REQ ID	Requirement Definition
REQ-01	**Issuance of unique agent identity.** Register each AI agent as a unique non-human identity (NHI), separate from human and shared service accounts, and manage its credentials, privileges, purpose, and execution history.
REQ-02	**Agent credential lifecycle management.** Issue short-lived, task-scoped credentials, revoke them after use, and continuously re-authenticate long-lived sessions.
REQ-03	**Delegation boundary verification and privilege ceiling.** Verify that delegated privileges remain within the original intent and do not exceed those of the delegating human or agent.
REQ-04	**Action–intent binding.** Cryptographically bind tool invocations and actions to user intent and the current workflow stage to prevent intent drift across delegation chains.
REQ-05	**Mutual authentication between agents.** Require mutual authentication for agent-to-agent interactions and block unapproved cross-agent privilege delegation.
REQ-06	**Agent execution environment isolation.** Run agents, tools, and code in isolated sandboxes, and separate or reset session states to prevent privilege escalation and cross-session leakage.
REQ-07	**Runtime environment integrity & compliance monitoring.** Continuously verify runtime compliance, including image signatures, non-root execution, and network restrictions, and isolate agents operating in compromised environments.
REQ-08	**AI software supply chain verification.** Verify the provenance of models, tools, prompts, agent descriptors, and MCP servers using signatures and attestations such as SBOM/AIBOM, and reject unverified components.
REQ-09	**Emergency invalidation and isolation response.** Provide mechanisms such as kill switches to disable and isolate compromised agents, credentials, models, tools, runtimes, and memory/RAG sources, and re-verify their integrity at runtime.
REQ-10	**Protection of agent communication integrity.** Digitally sign inter-agent messages and verify their payloads and context to detect tampering and hidden command injection.
REQ-11	**Protocol version and schema verification.** Enforce approved MCP and A2A protocol versions and typed schemas, and reject downgrade attempts and unrecognized messages.
REQ-12	**External entity trust verification.** Verify the identity, integrity, and authorized scope of external APIs, MCP servers, and external agents, and restrict egress through destination allowlists.
REQ-13	**Isolated processing of untrusted external data.** Treat external sources, including web pages, emails, and documents, as untrusted inputs and apply validation and prompt-injection defenses.
REQ-14	**Explicit definition of Action Space.** Define each agent’s permitted, conditional, and prohibited actions, and require policy enforcement points (PEPs) to block out-of-scope actions before execution.
REQ-15	**Tool-specific least-privilege profiles.** Define tool-specific profiles for access scope, invocation frequency, and egress, and restrict each tool to the approved privileges and data scope.
REQ-16	**Pre-execution intent verification.** Use an Intent Gate to verify that tool invocations match the current task context and to validate arguments, schemas, and parameters before execution.
REQ-17	**Action risk-based differentiated approval.** Classify actions by reversibility, real-time impact, data sensitivity, external transfer, privilege, and autonomy, and apply human approval or enhanced verification to high-risk actions.
REQ-18	**Just-in-time privilege granting.** Grant tool privileges only when required, revoke them after use, and restrict execution according to current risk.
REQ-19	**AI-generated code execution control.** Separate code generation from execution, apply verification such as static analysis and taint tracking, and restrict execution to approved environments.
REQ-20	**Action rate and frequency limiting.** Limit API calls and high-cost operations, and detect abnormal execution rates or tool chaining to prevent overload and cascading execution.
REQ-21	**Validation of memory write content.** Inspect writes to long-term memory and RAG stores for malicious or anomalous content, and separate memory across sessions to prevent unauthorized knowledge transfer.
REQ-22	**Memory provenance and trust management.** Record the provenance of memory updates, assign trust scores to entries, and expire or decay unverified memory to limit persistent contamination.
REQ-23	**Context integrity and information flow tracking.** Track data flows from context loading through model and tool invocation to output generation, and detect unauthorized movement of sensitive data.
REQ-24	**Context-aware data access control.** Determine access scope from identity, privilege, classification, task purpose, and execution context, and block excessive, unauthorized, or out-of-purpose access.
REQ-25	**Prevention of reinjection of self-generated outputs.** Prevent unverified agent outputs from being reintroduced into trusted memory or context to limit self-reinforcing poisoning and cascading hallucinations.
REQ-26	**Cryptographic audit logs.** Preserve agent actions, tool invocations, and memory access in signed, tamper-resistant logs, and link delegation chains through a common trace ID.
REQ-27	**Agent behavioral baseline and anomaly detection.** Establish behavioral baselines, detect goal drift, abnormal tool use, privilege escalation, and cross-agent anomalies, and trigger isolation or kill-switch responses when necessary.
REQ-28	**Autonomy level management and deployment approval.** Require security review, approval of the Autonomy Level, and assignment of an accountable owner before deployment, and adjust autonomy according to trust and operational history.
REQ-29	**HITL optimization and approval fatigue prevention.** Prioritize human review by risk, automate low-risk tasks, and focus oversight on high-risk actions to reduce approval fatigue and automation bias.
REQ-30	**Multi-agent pipeline trust management.** Monitor each agent’s actions and trust state, and suspend the pipeline or isolate an agent when anomalous behavior or trust degradation is detected.
REQ-31	**Agent lifecycle governance.** Govern agent creation, deployment, operation, and retirement, conduct periodic reviews, and verify the deletion of identities, credentials, memory, and delegation relationships at retirement.

## Appendix B. Maturity Criteria for Security Functions by Pillar

Each security Function is assessed across four levels: Traditional, Initial, Advanced, and Optimal. Traditional does not mean the absence of control. For extended Functions, it refers to a state in which existing CISA ZTMM controls for human users and general resources are applied, but agent-specific controls have not yet been introduced. For new Functions, it refers to a state in which the control is not separately performed because it has no direct lineage in CISA. The levels generally progress along the axis of manual and static controls (Initial), policy-based automated controls (Advanced), and dynamic, adaptive, and integrated controls (Optimal).

### Appendix B.1. Identity

**Table A2:** Maturity-level criteria for Identity Pillar.

Level	Assessment Criteria
**ID-1 Identity stores**	
Traditional	Agents use human or shared service accounts, without separate identities or an agent inventory.
Initial	Unique identifiers are manually issued to some agents, but registration is inconsistent and ownership, privilege scope, and purpose are not centrally managed.
Advanced	All agents are registered as unique non-human identities (NHIs), with ownership, privilege scope, purpose, and execution history centrally managed. Unauthorized agents are detected.
Optimal	Identity stores are synchronized with execution environments in real time. Agent registration and deprovisioning are automated, and unauthorized or ghost agents are blocked.
**ID-2 Authentication**	
Traditional	Agents are not separate authentication subjects and rely on static API keys or shared secrets. Agent-to-agent authentication is absent.
Initial	Agents receive individual credentials, but credentials remain static and long-lived. External federation is manual or absent.
Advanced	Agent identity is verified for access to resources, tools, and other agents. Mutual authentication, federation, and credential lifecycle controls are enforced.
Optimal	Authentication strength adapts to risk and context, while inter-agent and cross-organizational trust are automatically governed by policy.
**ID-3 Access management**	
Traditional	Agents receive broad, static privileges that remain active without timely review or revocation.
Initial	Role-based restrictions are introduced, but privileges remain pre-granted and persistent, without delegation privilege ceilings.
Advanced	Privileges are granted through just-in-time (JIT) access and revoked after use. Delegated privileges cannot exceed those of the delegating principal.
Optimal	Privilege, token, and session scopes are dynamically adjusted based on real-time risk and automatically reduced or revoked upon anomalies.
**ID-4 Risk assessment**	
Traditional	Access decisions rely on static rules without considering agent behavior or state.
Initial	Risk assessment uses limited login factors, but excludes execution context and behavioral history.
Advanced	Risk scores combine behavioral trajectory, task context, credential state, session metadata, and execution environment state for authorization decisions.
Optimal	Risk is continuously recalculated, privileges are adjusted during sessions, and the risk model is refined using incidents, anomalies, and audit feedback.
**ID-5 Delegation chain control**	
Traditional	Agent delegation is not separately controlled, with no limits on boundaries, re-delegation, or chain depth.
Initial	Delegation relationships are recorded, but boundaries, re-delegation, and validity periods are not enforced.
Advanced	Delegation boundaries, re-delegation permissions, chain depth, and validity periods are enforced by policy.
Optimal	Delegation chains are verified in real time using a common trace ID. Anomalous delegation is blocked, and privileges expire when tasks complete.
**ID-6 Visibility & Analytics**	
Traditional	Agent identity, authentication, and privilege events are mixed with human account logs and cannot be attributed to individual agents.
Initial	Agent authentication and access logs are collected, but delegation is not tracked and analysis remains retrospective and manual.
Advanced	Identity use, authentication, privilege exercise, and delegation are recorded and correlated through a common trace ID. Identity and privilege anomalies are analyzed.
Optimal	Identity and privilege anomalies are correlated in real time, and behavioral baselines are continuously updated to detect unknown patterns.
**ID-7 Automation & Orchestration**	
Traditional	Credential theft and anomalous authentication are handled manually, resulting in delayed response.
Initial	Limited actions, such as account locking, are automated without agent-wide orchestration.
Advanced	Credential invalidation, privilege revocation, and agent isolation are automatically orchestrated when identity or delegation anomalies are detected.
Optimal	Detection and response operate as a real-time closed loop, delegation chains are blocked end-to-end, and playbooks are refined using incident history.
**ID-8 Governance**	
Traditional	Human identity policies are applied to agents without agent-specific credential policies or accountable ownership.
Initial	Agent identity issuance criteria are documented, but credential rotation, retirement, and review processes remain inconsistent.
Advanced	Policies for identity issuance, ownership, credential lifecycle, and retirement are established and regularly reviewed.
Optimal	Identity governance covers the full agent lifecycle, policy violations are automatically detected and corrected, and audit findings inform policy updates.

### Appendix B.2. Devices and Runtime Environments

**Table A3:** Maturity-level criteria for Devices & Runtime Environments Pillar.

Level	Assessment Criteria
**DE-1 Execution foundation policy enforcement**	
Traditional	Physical devices and servers are checked for compliance, but agent runtime integrity is not separately verified.
Initial	Integrity checks are applied to some runtime environments, but remain manual and irregular, without automated response.
Advanced	Runtime environments and physical execution devices are verified for integrity, and degraded foundations are restricted or isolated.
Optimal	Integrity is continuously verified at execution time, and compromised foundations trigger real-time blocking or workload relocation.
**DE-2 Asset inventory**	
Traditional	Physical devices and servers are inventoried, but agent containers, VMs, and serverless environments are not separately tracked.
Initial	Some runtime environments are inventoried, but dynamic environments and their owning agents are not consistently tracked.
Advanced	Hosts, devices, and runtime environments have unique identifiers, and dynamic environments are linked to their agents and dependencies.
Optimal	Inventories synchronize with orchestration platforms in real time. Registration and deprovisioning are automated, and ghost environments are detected.
**DE-3 Resource access control**	
Traditional	Device-level controls exist, but agent, tool, and code execution lack isolation and session separation.
Initial	Some execution is isolated, but session state separation is incomplete and resource limits remain static.
Advanced	Resource access is policy-restricted, execution is isolated, and state is separated and reset between sessions.
Optimal	Isolation and resource access adapt to action risk and context, while cross-session leakage is automatically detected and blocked.
**DE-4 Device and runtime environment threat protection**	
Traditional	Endpoint protection exists, but runtime threats such as sandbox escape and unauthorized system calls are not specifically addressed.
Initial	Runtime threat detection is introduced but remains signature-based, reactive, and limited in detecting constraint bypass.
Advanced	Sandbox escape, unauthorized system calls, runtime constraint bypass, and execution-device compromise are detected and blocked.
Optimal	Behavior-based detection operates in real time, compromised environments are automatically isolated, and detection rules are continuously refined.
**DE-5 Visibility & Analytics**	
Traditional	Device and server logs are collected, but runtime state and configuration changes are not separately identifiable.
Initial	Runtime logs are collected, but analysis remains manual and retrospective, without anomaly detection.
Advanced	Runtime state, configuration changes, and anomalous execution are recorded and analyzed.
Optimal	Runtime anomalies are detected and correlated in real time, and normal-state baselines are continuously updated.
**DE-6 Automation & Orchestration**	
Traditional	Responses to execution foundation compromise are fully manual.
Initial	Limited actions, such as restart, are automated without environment-wide orchestration.
Advanced	Integrity degradation or compromise triggers automated isolation, regeneration, and blocking of agent execution.
Optimal	Detection and response form a real-time closed loop; compromised environments are automatically regenerated; and playbooks are continuously refined.
**DE-7 Governance**	
Traditional	Device and server policies exist, but agent runtime approval, image, and isolation policies are absent.
Initial	Runtime approval criteria are documented, but image and isolation policies are inconsistently enforced.
Advanced	Runtime approval criteria, permitted images, and isolation policies are established and regularly reviewed.
Optimal	Governance is integrated with deployment pipelines, non-compliant deployments are automatically blocked, and audit findings inform policy updates.

### Appendix B.3. Networks

**Table A4:** Maturity-level criteria for Networks Pillar.

Level	Assessment Criteria
**NW-1 Network segmentation**	
Traditional	Network segments exist, but agent communication is not separated by business function or trust level.
Initial	Agent traffic is partially separated using static rules that do not consider identity or task purpose.
Advanced	Communication paths are segmented by function and trust level, with access controlled by agent identity, task purpose, and execution context.
Optimal	Segmentation dynamically adapts to trust state and context, and anomalous communication paths are automatically blocked.
**NW-2 Network traffic management**	
Traditional	Traffic filtering exists, but agent communications with external APIs, MCP servers, and external agents lack dedicated egress controls and rate limits.
Initial	Static egress allowlists and rate limits are partially applied, while external entity verification remains manual.
Advanced	External entities are verified, egress allowlists are enforced, and external or high-cost communications are rate-limited.
Optimal	Egress controls and rate limits adapt to action risk and context, blocking unauthorized communication and resource exhaustion attempts.
**NW-3 Communication integrity protection**	
Traditional	Transport encryption is used, but agent protocols such as MCP and A2A lack message-level integrity verification.
Initial	Message signing is partially introduced, but schema and protocol version verification remain incomplete.
Advanced	Messages are signed and verified for controlled agent protocols. Tampering, schema manipulation, downgrade attempts, and unauthorized injection are blocked, and approved versions are enforced.
Optimal	End-to-end protocol integrity is verified in real time, anomalous messages are automatically blocked, and protocol policies are continuously updated.
**NW-4 Visibility & Analytics**	
Traditional	Network logs are collected, but agent external communication and egress activity are not separately identifiable.
Initial	Agent communication logs are collected, but analysis remains manual and retrospective, without anomalous destination detection.
Advanced	External communication, egress activity, and protocol usage are recorded and analyzed for anomalous destinations and communication patterns.
Optimal	Communication anomalies are detected and correlated in real time, and normal communication baselines are continuously updated.
**NW-5 Automation & Orchestration**	
Traditional	Unauthorized communication is handled entirely through manual response.
Initial	Limited blocking is automated, but responses are not orchestrated across agent communication paths.
Advanced	Unauthorized egress or anomalous communication triggers automated connection blocking and isolation.
Optimal	Detection and blocking operate as a real-time closed loop, and response policies are continuously refined.
**NW-6 Governance**	
Traditional	General network policies exist, but agent-specific destination and protocol policies are absent.
Initial	Agent communication policies are documented, but enforcement and review are inconsistent.
Advanced	Policies for permitted destinations, protocols, and segmentation are established and regularly reviewed.
Optimal	Communication policies are automatically enforced and audited, with violations automatically detected and corrected.

### Appendix B.4. Applications, Workloads and Agent Actions

**Table A5:** Maturity-level criteria for Applications, Workloads & Agent Actions Pillar.

Level	Assessment Criteria
**AW-1 Tool access management**	
Traditional	Application access controls exist, but agent tools lack allowlists and least-privilege profiles.
Initial	Major tools are allowlisted, but privilege, invocation frequency, and egress scope are not granularly defined.
Advanced	Tools are allowlisted with least-privilege profiles for access, invocation frequency, and egress. Unauthorized invocations are blocked.
Optimal	Tool privileges dynamically adapt to task context and risk, and anomalous use is automatically blocked.
**AW-2 Application threat protection**	
Traditional	General application protection exists, but agent-specific threats such as prompt injection and untrusted inputs are not addressed.
Initial	External input validation and prompt injection detection are partially applied using static rules.
Advanced	Untrusted external data is validated and isolated, while prompt injection, instruction tampering, jailbreaks, and adversarial inputs are detected and blocked.
Optimal	Detection is behavior-based and adaptive, with controls refined using new attacks, incidents, and audit findings.
**AW-3 AI software supply chain verification**	
Traditional	Deployment pipelines exist, but the provenance of models, tools, MCP components, and plugins is not verified.
Initial	Some component provenance is manually checked, but signatures, attestations, and AIBOM use are not systematic.
Advanced	Models, tools, MCP components, plugins, and workflows are verified through signatures and attestations. Unverified components are rejected.
Optimal	Supply chain verification is enforced during deployment and runtime, and compromised components are immediately invalidated.
**AW-4 Agent security testing and evaluation**	
Traditional	Application testing exists, but agent-specific adversarial testing is not performed.
Initial	Limited manual testing occurs before deployment, but it is irregular and unsystematic.
Advanced	Prompt injection, intent drift, tool misuse, and code execution risks are regularly tested before and after deployment, with results used for approval.
Optimal	Adversarial testing is continuously integrated into CI/CD and informs control and policy updates.
**AW-5 Action scope definition and enforcement**	
Traditional	Agent action scope is undefined, allowing any action within granted privileges.
Initial	Some prohibited actions are listed, but Action Space is not systematically structured or fully enforced before execution.
Advanced	Actions are classified as permitted, conditional, or prohibited across defined action types, and PEPs block out-of-scope actions before execution.
Optimal	Action Space dynamically adapts to context and risk, and violations are blocked and incorporated into policy updates.
**AW-6 Pre-execution intent verification**	
Traditional	Planner outputs and tool invocations are executed without prior verification.
Initial	Parameters are partially validated, but invocation intent is not checked against task context.
Advanced	Planner outputs are treated as untrusted, and invocation intent, arguments, schemas, and parameters are verified before execution.
Optimal	Intent is verified in real time, and detected intent drift is reflected in policy updates.
**AW-7 Differentiated action-risk control**	
Traditional	All agent actions receive the same controls regardless of risk.
Initial	Some high-risk actions require approval, but risk criteria remain static and incomplete.
Advanced	Actions are assessed using reversibility, immediacy, data sensitivity, external transmission, privilege, autonomy, and impact scope. High-risk actions require HITL or enhanced verification.
Optimal	Action risk is assessed in real time, and control strength dynamically adapts to context and agent trust state.
**AW-8 AI-generated code execution gate**	
Traditional	Agent-generated code is executed without verification or separation between generation and execution.
Initial	Some code runs in isolated environments, but no systematic execution gate exists.
Advanced	Generation and execution are separated, and code runs only in restricted environments after checks for risky commands, external calls, and privilege escalation.
Optimal	Static and dynamic analysis are integrated into the gate, with execution automatically allowed or blocked according to real-time risk.
**AW-9 Visibility & Analytics**	
Traditional	Application logs exist, but agent actions, tool invocations, and outcomes are not separately recorded.
Initial	Agent action logs are collected, but behavioral baselines and anomaly analysis are absent.
Advanced	Agent actions, tool invocations, and outcomes are recorded and analyzed for deviations such as goal drift and rogue-agent behavior.
Optimal	Action anomalies are detected and correlated in real time, and behavioral baselines are continuously updated.
**AW-10 Automation & Orchestration**	
Traditional	Responses to anomalous agent behavior are entirely manual.
Initial	Limited blocking is automated, but multi-agent pipeline responses are not orchestrated.
Advanced	Anomalies or compromise trigger automated action blocking, agent isolation, and emergency invalidation. Multi-agent pipelines are suspended or isolated to prevent cascading failures.
Optimal	Detection and response operate as a real-time closed loop, with automated cascading containment and continuously refined playbooks.
**AW-11 Governance**	
Traditional	Application policies exist, but agent Action Space and autonomy levels are not governed.
Initial	Action Space and autonomy approval procedures are introduced, but reassessment and HITL policies remain inconsistent.
Advanced	Policies cover Action Space approval, Autonomy Level assignment and reassessment, HITL prioritization, and approval-fatigue prevention.
Optimal	Governance incorporates operational history and trust state, dynamically adjusts autonomy, and automatically corrects policy violations.

### Appendix B.5. Data

**Table A6:** Maturity-level criteria for Data Pillar.

Level	Assessment Criteria
**DA-1 Data asset management**	
Traditional	Data inventories exist, but agent memory, RAG, and context data are not separately tracked.
Initial	Some agent data sources are inventoried, but memory, RAG assets, and information flows remain incomplete.
Advanced	Agent-accessed, generated, and stored data are inventoried and classified, and data flows across context loading, model and tool invocation, and output generation are tracked.
Optimal	Data inventories and flows are tracked in real time, and unauthorized sensitive-data paths are automatically blocked.
**DA-2 Context-aware data access**	
Traditional	Data access controls do not consider agent task purpose or execution context.
Initial	Role-based access is applied, but task context and trust state do not dynamically affect access.
Advanced	Access is restricted according to task purpose, execution context, and agent trust level.
Optimal	Access scope dynamically adapts to real-time context and risk, blocking excessive queries and unauthorized data combinations.
**DA-3 Memory integrity protection**	
Traditional	Agent memory and RAG stores lack write-integrity checks and session isolation.
Initial	Some memory writes are inspected, but controls are static and cross-session separation is incomplete.
Advanced	Memory, RAG, and model-output writes are inspected for poisoning, and memory is isolated across sessions.
Optimal	Writes are verified in real time using policy and context, and suspected poisoning is automatically isolated or blocked.
**DA-4 Memory provenance and trust management**	
Traditional	Memory provenance and trust are not managed, and all entries are treated equally.
Initial	Provenance is recorded for some entries, but trust weighting and expiration are absent.
Advanced	Memory updates require provenance, entries are trust-weighted, and unverified memory expires or decays.
Optimal	Trust is continuously reassessed, and low-trust or poisoned memory is automatically isolated and discarded.
**DA-5 Context trust boundary isolation**	
Traditional	Trusted and untrusted context data are mixed without explicit boundaries.
Initial	Some external data is labeled, but trust-boundary isolation remains incomplete.
Advanced	Trusted and untrusted context data are separated, and unverified data cannot influence decisions or actions.
Optimal	Context trust boundaries are enforced in real time, and malicious influence from untrusted data is automatically blocked.
**DA-6 Output re-ingestion control**	
Traditional	Agent outputs may be re-ingested into trusted memory or context without verification.
Initial	Re-ingestion is partially restricted, but provenance separation and verification remain incomplete.
Advanced	Agent outputs require verification before entering trusted memory or context, limiting self-reinforcing poisoning and cascading hallucinations.
Optimal	Re-ingestion is controlled in real time using provenance, and self-reinforcing poisoning patterns are automatically blocked.
**DA-7 Visibility & Analytics**	
Traditional	Data access logs exist, but memory writes and RAG references are not separately recorded.
Initial	Agent data access is logged, but poisoning indicators are not analyzed.
Advanced	Data access, memory writes, and RAG references are recorded and analyzed for anomalous access and poisoning.
Optimal	Data anomalies are correlated in real time, and detection rules are refined using incidents and audit findings.
**DA-8 Automation & Orchestration**	
Traditional	Responses to data poisoning are entirely manual.
Initial	Limited isolation is automated, but poisoned-content removal and session termination are not orchestrated.
Advanced	Poisoning or anomalous access triggers automated source isolation, session termination, content removal, and access blocking.
Optimal	Detection and response operate as a real-time closed loop, automatically containing poisoning spread and refining playbooks.
**DA-9 Governance**	
Traditional	General data policies exist, but agent access, memory retention, and RAG sources lack specific governance.
Initial	Agent data and RAG source policies are documented, but enforcement and review are inconsistent.
Advanced	Policies for agent data access, memory retention, and RAG source approval are established and regularly reviewed.
Optimal	Data policies are automatically enforced and audited, with violations automatically detected and corrected.
