# Supplementary material for: An Action-Centric Zero Trust Maturity Model for Agentic AI Environments
Source: Sensors (Basel). 2026 Aug 17;26(16):5205. doi: 10.3390/s26165205 (PMC13517456; doi:10.3390/s26165205)
Supplement: Supplementary file 1 [file sensors-26-05205-s001.zip › File S1. Derivation Codebook and Requirement.pdf]

Derivation Codebook and Requirement-to-Pillar Coding Record

An Action-Centric Zero Trust Maturity Model for Agentic AI Environments

This supplement documents the coding framework used to derive AI-ZTMM and the final record produced through its application. Section 1 explains how the threat areas, security requirements, and security Functions were derived. Section 2 presents the codebook used to assign each requirement to the AI-ZTMM Pillars and Cross-cutting Capabilities. Section 3 records the coding decisions for all thirty-one requirements, while Section 4 links those requirements to their supporting sources. The coding procedure and revision history are described in Section 5.

It was subsequently revised through six expert review sessions conducted between January and June 2026. Whenever a decision rule was changed, the requirements affected by that rule were reviewed and coded again. The codebook and coding record presented here are the final versions resulting from that process.

1. Derivation Criteria

The model is built as a one-way chain: threat area → security requirement → security Function → Pillar. Each step has an admission criterion, and an element is admitted only if it satisfies the criterion of its own step and is linked to at least one element of the preceding step. The criteria are stated below in the order in which they were applied.

1.1 Threat areas

The threat areas were not created specifically for this study. Instead, existing public taxonomies of Agentic AI threats were reviewed and consolidated. A threat area was retained only when all three conditions below were met.

Condition	Criterion
T1. Traceable to a published taxonomy	The threat area had to correspond to one or more entries in a publicly available Agentic AI threat taxonomy. Threats proposed only by the present study were not included.
T2. Specific to agentic systems	The threat had to arise from characteristics such as autonomy, tool invocation, delegation, or persistent memory. General software threats that apply without any Agentic AI-specific modification were excluded.
T3. Distinguishable from adjacent areas	The threat area had to be distinguishable from other areas by its primary attack object. Candidate areas addressing the same object were consolidated.

Application of these criteria yielded the following ten threat areas.

ID	Threat area
ASI-1	Agent Goal Hijack
ASI-2	Tool Misuse & Exploitation
ASI-3	Identity & Privilege Abuse
ASI-4	Agentic Supply Chain Vulnerabilities

ASI-5	Unexpected Code Execution
ASI-6	Memory & Context Poisoning
ASI-7	Insecure Inter-Agent Communication
ASI-8	Cascading Failures
ASI-9	Human-Agent Trust Exploitation
ASI-10	Rogue Agent

1.2 Security requirements

Security requirements were extracted from the source set used for requirement derivation and summarized in Section 4. Four rules were used to determine whether an extracted control should be retained as a requirement.

These rules produced thirty-one security requirements, identified as REQ-01 through REQ-31.

Condition	Criterion
R1. Extraction unit	The extraction unit was the smallest independently meaningful control: one control, mitigation measure, or security principle stated in a source. A passage containing several controls was divided before coding.
R2. Consolidation	Controls expressing the same control objective were combined into one requirement, even when different sources used different terminology. All supporting sources were retained in the consolidated record.
R3. Agentic AI specificity	A requirement had to address one of the threat areas identified in Section 1.1 and either introduce a control not ordinarily required in conventional LLM or IT environments, or require a materially different implementation. General IT controls that could be applied without modification were excluded.
R4. Documentation	Each requirement had to include its supporting sources, related threat area, control objective, and Pillar-assignment rationale. Items that could not be documented on all four points were returned to the extraction stage.

1.3 Security Functions

Security Functions are the assessable units of AI-ZTMM. A Function was defined only after its associated requirement had been assigned to a Pillar or Cross-cutting Capability. Three criteria were applied.

Condition	Criterion
F1. Requirement linkage	Each Function had to implement at least one accepted security requirement. A Function without a requirement-level basis was not included.
F2. Assessability	The Function had to be assessable at the four maturity levels using observable evidence. Broad statements of intent that could not be translated into maturity criteria were excluded.
F3. Avoidance of unnecessary restatement	Controls that apply to agents in the same way that they apply to ordinary users and systems remain within the CISA ZTMM and were not repeated. A Function was classified as Extended when an existing CISA Function required modification for agents, and as New when no equivalent Function existed.

This process resulted in forty-one Functions: twenty-six Pillar-specific Functions and fifteen cross-cutting Function slots. The latter comprise the three Cross-cutting Capabilities instantiated within each of the five Pillars.

1.4 Integrity of the Derivation Chain

Two checks were applied to the completed derivation chain, and the final coding record satisfied both conditions.

Condition	Criterion
C1. No orphaned elements	Every threat area had to be addressed by at least one requirement. Every requirement had to be assigned to a Pillar or Cross-cutting Capability and implemented by at least one Function. Each Function, in turn, had to be traceable to a requirement and a threat area.
C2. One-way derivation	Links had to proceed in one direction, from threat areas to requirements and then to assigned Pillars, Capabilities, and Functions. A Function could not introduce a control objective that was absent from the requirement on which it was based.

2. Pillar Assignment Codebook

The codebook contains one entry for each of the five Pillars and three Cross-cutting Capabilities. Each entry identifies the control object covered by the category, the ordinary assignment rule, and the boundary rule used when a requirement could reasonably be placed in an adjacent category. The worked examples in Section 2.4 illustrate how those rules were applied in practice.

2.1 Coding unit and permitted codes

The coding unit was one consolidated security requirement, from REQ-01 to REQ-31. A requirement was coded as a whole; individual source controls that had already been consolidated were not coded separately.

Code	Assignment Target
P1	Identity
P2	Devices & Runtime Environments
P3	Networks
P4	Applications, Workloads & Agent Actions
P5	Data
C1	Visibility & Analytics
C2	Automation & Orchestration
C3	Governance

A requirement was assigned more than one code only when its implementation required controls at separate control points. Merely affecting several Pillars was not sufficient. Where one control was enforced at a single point but produced effects elsewhere, only the code corresponding to the direct control object was assigned.

2.2 Decision procedure

The assignment procedure was applied as follows.

1. Identify the requirement's direct control object—that is, the object on which the control operates rather than the asset it ultimately protects.
2. Select the Pillar whose assignment criterion best matches that object.
3. When two Pillars appear applicable, compare the boundary rules for both and record the rule used to resolve the case.
4. Where the principal purpose is observation, automated response, or policy governance, assign C1, C2, or C3 instead of, or in addition to, a Pillar.
5. Do not use multiple codes simply because a requirement has consequences across several Pillars.
6. Use multiple codes only when separate implementation is required at distinct control points, and record the relevant control points.

2.3 Codebook entries

2.3.1. P1 – Identity

Field	Content
Classification target	The identity, credentials, and delegation relationships of the subject that accesses resources or performs actions
Agentic AI perspective	AI agents are treated as independent identity subjects. They are registered and managed as unique non-human identities rather than as extensions of human accounts or shared service accounts.
Assignment criterion	Assign the requirement to Identity when its direct control object is the identification, authentication, authorization, or delegation of the acting subject.
Boundary decision criterion	The granting and delegation of privilege belong to Identity. The actions subsequently performed with that privilege belong to Applications, Workloads & Agent Actions. For example, issuing a privilege when it is needed is an Identity control, whereas limiting what an invoked tool may do is an action-layer control.
Representative Functions	ID-1 Identity stores; ID-2 Authentication; ID-3 Access management; ID-4 Risk assessment; ID-5 Delegation chain control
Corresponding threats	ASI-3 Identity & Privilege Abuse; ASI-7 Insecure Inter-Agent Communication
Supporting references	S2, S5, S9, R1, R7, R12

2.3.2 P2 – Devices & Runtime Environments

Field	Content
Classification target	The execution foundation on which agents run, including physical devices, containers, virtual machines, and serverless runtimes
Agentic AI perspective	Trust in an agent's actions depends on the integrity of its execution foundation. Once that foundation is compromised, actions performed above it cannot be treated as trustworthy.
Assignment criterion	Assign the requirement to this Pillar when the control acts on the integrity, isolation, inventory, or security state of the environment in which an action is executed.
Boundary decision criterion	For AI-generated code, isolation of the execution environment belongs to Devices & Runtime Environments, while the policy decision permitting execution belongs to Applications, Workloads & Agent Actions. In supply-chain cases, the integrity of the build or execution foundation belongs here, whereas provenance checks for models, tools, and other AI software components belong to the application layer.
Representative Functions	DE-1 Execution foundation policy enforcement; DE-2 Asset inventory; DE-3 Resource access control; DE-4 Threat protection
Corresponding threats	ASI-4 Agentic Supply Chain Vulnerabilities; ASI-5 Unexpected Code Execution
Supporting references	S2, S5, S9, R4

2.3.3 P3 — Networks

Field	Content
Classification target	Communication paths between agents and external entities, including external APIs, MCP servers, and agents operated by other organizations
Agentic AI perspective	Agents may select communication counterparts dynamically at runtime. As a result, their trust boundaries are less fixed than those of conventional applications.
Assignment	Assign the requirement to Networks when it verifies or restricts the

criterion	communication counterparty, destination, protocol, or path.
Boundary decision criterion	Verification of an external endpoint and restriction of outbound destinations belong to Networks. Validation of the content returned by that endpoint belongs to Applications, Workloads & Agent Actions. The assignment follows the object of the control rather than the direction in which traffic moves.
Representative Functions	NW-1 Network segmentation; NW-2 Network traffic management; NW-3 Communication integrity protection
Corresponding threats	ASI-7 Insecure Inter-Agent Communication; ASI-2 Tool Misuse & Exploitation
Supporting references	S2, S9, R6, R15

2.3.4 P4 – Applications, Workloads & Agent Actions

Field	Content
Classification target	The actions an agent performs through applications, tools, APIs, and code execution environments
Agentic AI perspective	What the agent does after access has been granted is the central assessment target. This is where the model extends access-centric assessment to action-centric verification
Assignment criterion	The control object of the requirement is the scope, intent, or risk level of the action itself
Boundary decision rule	The privilege to invoke a tool belongs to Identity; the set of tools invocable and the conditions of invocation belong to AW. The privilege to read data belongs to Data (REQ-24); the act of transmitting data outward belongs to AW and Networks
Include	Binding of a tool invocation to user intent (REQ-04); Provenance verification of models, tools, and MCP components (REQ-08); Validation of untrusted external content before it enters reasoning (REQ-13); Definition and enforcement of Action Space (REQ-14); Tool-specific least-privilege profiles (REQ-15); Pre-execution intent verification (REQ-16); Risk-based differentiated approval (REQ-17); The gate separating code generation from execution (REQ-19)
Exclude	Possession or delegation of credentials → P1; Integrity of the runtime the agent executes on → P2; Communication-path and destination restrictions → P3; Integrity of stored memory → P5
Representative Functions	AW-1 Tool access management, AW-2 Application threat protection, AW-3 AI software supply chain verification, AW-5 Action scope definition, AW-6 Pre-execution intent verification, AW-7 Differentiated action-risk control, AW-8 AI-generated code execution gate

2.3.5. P5 – Data

Field	Content
Classification target	Data that informs the agent's reasoning and actions, particularly memory, RAG stores, and execution context
Agentic AI perspective	Data is not only a resource to be protected; it also shapes the agent's decisions. Poisoned memory may persist across sessions and repeatedly influence later actions.
Assignment criterion	Assign the requirement to Data when it governs the integrity, provenance, trust status, accessibility, or retention of information that the agent remembers or consults.
Boundary decision criterion	Validation of external content when it first enters the application belongs to Applications, Workloads & Agent Actions. Its storage and isolation within context or memory belong to Data. Outbound transmission of data is addressed by the action and network layers.

Representative Functions	DA-1 Data asset management; DA-2 Context-aware data access; DA-3 Memory integrity protection; DA-4 Memory provenance and trust management; DA-5 Context trust boundary isolation; DA-6 Output re-ingestion control
Corresponding threats	ASI-6 Memory & Context Poisoning; ASI-1 Agent Goal Hijack; ASI-8 Cascading Failures
Supporting references	S2, S3, S9, R2, R23

2.3.6. C1 – Visibility & Analytics

Field	Content
Classification target	Recording and analysis of agent actions, delegation, communications, runtime activity, and data flows
Assignment criterion	Use C1 when observation or analysis is itself the main control purpose and the requirement must operate across more than one control domain.
Boundary decision criterion	Visibility & Analytics is not an independent Pillar. The applicable Function slot is determined by the Pillar that owns the observed object. A requirement is treated as cross-cutting when equivalent observation is required in multiple Pillars; observation confined to one Pillar remains assigned to that Pillar.
Representative Functions	ID-6, DE-5, NW-4, AW-9, DA-7
Corresponding threats	ASI-8 Cascading Failures; ASI-9 Human-Agent Trust Exploitation; ASI-10 Rogue Agent
Supporting references	S2, S3, S9

2.3.7 C2 – Automation & Orchestration

Field	Content
Classification target	Automated coordination of isolation, blocking, revocation, invalidation, and related response actions
Assignment criterion	Use C2 when the requirement concerns the automated response that follows detection and the response must be available across multiple control domains.
Boundary decision criterion	Detection and analysis belong to Visibility & Analytics. The action taken after detection belongs to Automation & Orchestration. The applicable Function slot depends on the Pillar responsible for the response target.
Representative Functions	ID-7, DE-6, NW-5, AW-10, DA-8
Corresponding threats	ASI-4, ASI-6, ASI-8, ASI-10
Supporting references	S2, S6, S9, R13, R18

2.3.8. C3 – Governance

Field	Content
Classification target	Policies governing agent deployment, Autonomy Levels, lifecycle management, ownership, and human-in-the-loop arrangements
Assignment criterion	Use C3 when the requirement primarily concerns the establishment, approval, ownership, or periodic review of policy rather than direct technical enforcement.
Boundary decision criterion	Establishing a policy belongs to Governance; enforcing that policy belongs to the relevant Pillar. For example, defining an Autonomy Level policy is a Governance matter, while applying different controls to actions according to autonomy is handled within Applications, Workloads & Agent Actions.
Representative Functions	ID-8, DE-7, NW-6, AW-11, DA-9
Corresponding threats	ASI-3, ASI-9, ASI-10
Supporting references	S3, S5, S6

3. Coding Record

Each subsection below records one requirement. “Sources” refers to the source keys defined in Section 4. “Threat area” identifies the threat or threats addressed by the requirement. The assignment rationale explains why the requirement was placed in the selected category. A boundary rule is recorded only when the requirement lay close to an adjacent category.

REQ-01 Issuance of unique agent identity

Field	Content
Control objective	Register each AI agent as a distinct non-human identity, separate from human users and shared service accounts, and maintain information on its credentials, privileges, purpose, ownership, and execution history.
Sources	S2, R1, S5
Threat area	ASI-3
Assigned code	P1
Applied Pillar(s)	Identity
Function(s)	ID-1
Assignment rationale	The requirement governs registration of the acting subject itself and therefore falls under the Identity criterion of determining who or what is acting.

REQ-02 Agent credential lifecycle management

Field	Content
Control objective	Issue short-lived and task-scoped credentials, revoke them after use, and re-authenticate long-running sessions as their context or risk changes.
Sources	S9, S2, R1
Threat area	ASI-3
Assigned code	P1
Applied Pillar(s)	Identity
Function(s)	ID-2
Assignment rationale	The control acts on credentials bound to an agent identity rather than on the resource eventually accessed with those credentials.

REQ-03 Delegation boundary verification and privilege ceiling

Field	Content
Control objective	Confirm that delegated privileges remain within the original task and intent, and prevent a receiving agent from obtaining privileges beyond those held by the delegating human or agent.
Sources	S5, S9, R7
Threat area	ASI-3
Assigned code	P1
Applied Pillar(s)	Identity
Function(s)	ID-3, ID-5
Assignment rationale	Delegation transfers privileges between subjects. The requirement limits the privileges that may be held rather than the actions later performed with them.

REQ-04 Action-intent binding

Field	Content
Control objective	Bind tool invocations and other actions to the originating user intent and current workflow stage, using cryptographic or equivalent verifiable mechanisms where

	appropriate, so that intent cannot silently drift across delegation chains.
Sources	S2, R7, S9
Threat area	ASI-1, ASI-3
Assigned code	P4
Applied Pillar(s)	Applications, Workloads & Agent Actions
Function(s)	AW-6
Assignment rationale	The binding is checked when the action or tool invocation is proposed. Its direct object is therefore the action, not the identity holding the underlying privilege.

REQ-05 Mutual authentication between agents

Field	Content
Control objective	Require mutual authentication before agent-to-agent interaction and prevent unapproved transfer or delegation of privileges between agents.
Sources	S9, S2, R12
Threat area	ASI-3, ASI-7
Assigned code	P1
Applied Pillar(s)	Identity
Function(s)	ID-2
Assignment rationale	Mutual authentication establishes who the counterpart agent is before information or authority is exchanged.

REQ-06 Agent execution environment isolation

Field	Content
Control objective	Execute agents, tools, and generated code in isolated environments, while separating or resetting session state to reduce privilege escalation, cross-session leakage, and unintended persistence.
Sources	S9, S2, R4
Threat area	ASI-2, ASI-3, ASI-5
Assigned code	P2
Applied Pillar(s)	Devices & Runtime Environments
Function(s)	DE-2, DE-3
Assignment rationale	Sandboxing and state separation operate directly on the execution foundation in which the agent runs.

REQ-07 Runtime environment integrity & compliance monitoring

Field	Content
Control objective	Continuously verify runtime compliance, including image signatures, non-root execution, and network restrictions, and isolate agents operating in compromised environments.
Sources	S9, S5, R4
Threat area	ASI-4, ASI-5
Pillar(s)	Devices & Runtime Environments
Function(s)	DE-1, DE-2, DE-4
Assignment rationale	Image signatures, non-root execution, and network restrictions are properties of the runtime, verified independently of any particular action.
Boundary rule applied	_

REQ-08 AI software supply chain verification

Field	Content
Control objective	Verify the provenance of models, tools, prompts, agent descriptors, and MCP servers using signatures and attestations such as SBOM/AIBOM, and reject unverified components.
Sources	S9, S2, R21
Threat area	ASI-4
Pillar(s)	Applications, Workloads & Agent Actions
Function(s)	AW-3
Assignment rationale	Provenance verification is applied to components at the point of registration and invocation, which is an application-layer control point.
Boundary rule applied	Devices / AW rule: the integrity of the runtime belongs to Devices; the integrity of the components an agent invokes belongs to AW.

REQ-09 Emergency invalidation and isolation response

Field	Content
Control objective	Provide mechanisms such as kill switches to disable and isolate compromised agents, credentials, models, tools, runtimes, and memory/RAG sources, and re-verify their integrity at runtime.
Sources	S9, S2, S6
Threat area	ASI-4, ASI-6, ASI-10
Pillar(s)	Identity; Devices & Runtime Environments; Networks; Applications, Workloads & Agent Actions; Data
Function(s)	ID-7, DE-6, NW-5, AW-10, DA-8
Assignment rationale	Emergency invalidation must be executable against whatever has been compromised, so the requirement is assigned to every Pillar and realised through the Automation & Orchestration slot of each.
Boundary rule applied	Cross-cutting rule: a requirement whose object is the automated response itself is assigned to A&O in every Pillar.

REQ-10 Protection of agent communication integrity

Field	Content
Control objective	Digitally sign inter-agent messages and verify their payloads and context to detect tampering and hidden command injection.
Sources	S9, S2, R6
Threat area	ASI-7
Pillar(s)	Networks
Function(s)	NW-3
Assignment rationale	Signing and payload verification protect the message while it crosses a trust boundary.
Boundary rule applied	-

REQ-11 Protocol version and schema verification

Field	Content
Control objective	Enforce approved MCP and A2A protocol versions and typed schemas, and reject downgrade attempts and unrecognized messages.
Sources	S9, S2
Threat area	ASI-7
Pillar(s)	Networks
Function(s)	NW-3

Assignment rationale	Protocol version and schema constraints are enforced on the communication channel before the payload reaches the agent.
Boundary rule applied	–

REQ-12 External entity trust verification

Field	Content
Control objective	Verify the identity, integrity, and authorized scope of external APIs, MCP servers, and external agents, and restrict egress through destination allowlists.
Sources	S9, S2, R15
Threat area	ASI-2, ASI-7
Pillar(s)	Networks
Function(s)	NW-1, NW-2
Assignment rationale	Verification of an external endpoint and egress allowlisting both govern communication with entities outside the trust boundary.
Boundary rule applied	Networks / AW rule: the decision to permit a destination belongs to Networks; the decision to permit a tool invocation belongs to AW.

REQ-13 Isolated processing of untrusted external data

Field	Content
Control objective	Treat external sources, including web pages, emails, and documents, as untrusted inputs and apply validation and prompt-injection defenses.
Sources	S2, S9
Threat area	ASI-1
Pillar(s)	Applications, Workloads & Agent Actions
Function(s)	AW-2
Assignment rationale	Untrusted external content is validated as an input to reasoning, which places the control at the application layer rather than on the channel.
Boundary rule applied	Networks / AW rule: the transport of external data belongs to Networks; the treatment of its content as untrusted input belongs to AW.

REQ-14 Explicit definition of Action Space

Field	Content
Control objective	Define each agent's permitted, conditional, and prohibited actions, and require policy enforcement points (PEPs) to block out-of-scope actions before execution.
Sources	S2, S3, R9, R29
Threat area	ASI-1, ASI-2
Pillar(s)	Applications, Workloads & Agent Actions
Function(s)	AW-5
Assignment rationale	Action Space defines the permitted range of actions and is enforced at a policy enforcement point before execution.
Boundary rule applied	–

REQ-15 Tool-specific least-privilege profiles

Field	Content
Control objective	Define tool-specific profiles for access scope, invocation frequency, and egress, and restrict each tool to the approved privileges and data scope.
Sources	S9, S2, R10
Threat area	ASI-2
Pillar(s)	Applications, Workloads & Agent Actions

Function(s)	AW-1
Assignment rationale	The profile constrains what a specific tool may do once invoked, which is the scope of the action rather than the privilege of the subject.
Boundary rule applied	Identity / AW rule: the scope of the action performed with a privilege belongs to AW.

REQ-16 Pre-execution intent verification

Field	Content
Control objective	Use an Intent Gate to verify that tool invocations match the current task context and to validate arguments, schemas, and parameters before execution.
Sources	S2, S9, R10, R11
Threat area	ASI-1, ASI-2
Pillar(s)	Applications, Workloads & Agent Actions
Function(s)	AW-6
Assignment rationale	The Intent Gate is evaluated between planning and execution, so its object is the invocation itself.
Boundary rule applied	–

REQ-17 Action risk-based differentiated approval

Field	Content
Control objective	Classify actions by reversibility, real-time impact, data sensitivity, external transfer, privilege, and autonomy, and apply human approval or enhanced verification to high-risk actions.
Sources	S9, S3
Threat area	ASI-1, ASI-2, ASI-9
Pillar(s)	Applications, Workloads & Agent Actions
Function(s)	AW-7
Assignment rationale	Differentiated approval is applied to an action according to its risk characteristics.
Boundary rule applied	–

REQ-18 Just-in-time privilege granting

Field	Content
Control objective	Grant tool privileges only when required, revoke them after use, and restrict execution according to current risk.
Sources	S9, S2, R6
Threat area	ASI-2, ASI-3
Pillar(s)	Identity
Function(s)	ID-3, ID-4
Assignment rationale	Just-in-time granting and revocation change the privilege held by the subject, independently of which tool is later used.
Boundary rule applied	Identity / AW rule: granting and delegation of privilege belong to Identity.

REQ-19 AI-generated code execution control

Field	Content
Control objective	Separate code generation from execution, apply verification such as static analysis and taint tracking, and restrict execution to approved environments.
Sources	S9, S2, R19
Threat area	ASI-5
Pillar(s)	Applications, Workloads & Agent Actions
Function(s)	AW-8
Assignment rationale	The execution gate separates generation from execution and is applied to the generated artefact before it runs.
Boundary rule applied	Devices / AW rule: the isolation of the environment belongs to Devices; the decision to execute a generated artefact belongs to AW.

REQ-20 Action rate and frequency limiting

Field	Content
Control objective	Limit API calls and high-cost operations, and detect abnormal execution rates or tool chaining to prevent overload and cascading execution.
Sources	S9, S2
Threat area	ASI-2, ASI-8
Pillar(s)	Networks
Function(s)	NW-2
Assignment rationale	Rate limiting is applied to outbound calls and to high-cost operations at the communication layer, where volume is observable.
Boundary rule applied	Networks / AW rule: limiting the rate of external calls belongs to Networks; limiting which calls are permitted belongs to AW.

REQ-21 Validation of memory write content

Field	Content
Control objective	Inspect writes to long-term memory and RAG stores for malicious or anomalous content, and separate memory across sessions to prevent unauthorized knowledge transfer.
Sources	S9, S2, R23
Threat area	ASI-6
Pillar(s)	Data
Function(s)	DA-3
Assignment rationale	Inspection of writes and cross-session separation act on the stored content itself.
Boundary rule applied	–

REQ-22 Memory provenance and trust management

Field	Content
Control objective	Record the provenance of memory updates, assign trust scores to entries, and expire or decay unverified memory to limit persistent contamination.
Sources	S9, S2, R23
Threat area	ASI-6
Pillar(s)	Data
Function(s)	DA-4
Assignment rationale	Provenance, trust weighting, and decay are attributes of stored entries.
Boundary rule applied	–

REQ-23 Context integrity and information flow tracking

Field	Content
Control objective	Track data flows from context loading through model and tool invocation to output generation, and detect unauthorized movement of sensitive data.
Sources	S2, S3, R23, S9
Threat area	ASI-1, ASI-6
Pillar(s)	Data
Function(s)	DA-1, DA-5
Assignment rationale	Flow tracking follows data from context loading to output generation, so the object is the data and its path.
Boundary rule applied	–

REQ-24 Context-aware data access control

Field	Content
Control objective	Determine access scope from identity, privilege, classification, task purpose, and execution context, and block excessive, unauthorized, or out-of-purpose access.
Sources	S9, S3, R2, R23
Threat area	ASI-3, ASI-6
Pillar(s)	Data
Function(s)	DA-2
Assignment rationale	Access scope is determined for a data request, which places the control on the data being accessed.
Boundary rule applied	Identity / Data rule: the privilege a subject holds belongs to Identity; the scope of data reachable under that privilege belongs to Data.

REQ-25 Prevention of reinjection of self-generated outputs

Field	Content
Control objective	Prevent unverified agent outputs from being reintroduced into trusted memory or context to limit self-reinforcing poisoning and cascading hallucinations.
Sources	S9, S2
Threat area	ASI-6, ASI-8
Pillar(s)	Data
Function(s)	DA-6
Assignment rationale	Re-ingestion control governs what may enter trusted memory or context.
Boundary rule applied	–

REQ-26 Cryptographic audit logs

Field	Content
Control objective	Preserve agent actions, tool invocations, and memory access in signed, tamper-resistant logs, and link delegation chains through a common trace ID.
Sources	S3, S9, S2
Threat area	ASI-8, ASI-9
Pillar(s)	Identity; Devices & Runtime Environments; Networks; Applications, Workloads & Agent Actions; Data
Function(s)	ID-6, DE-5, NW-4, AW-9, DA-7
Assignment rationale	Auditability is required for the objects of every Pillar, so the requirement is realised through the Visibility & Analytics slot of each.
Boundary rule	Cross-cutting rule: a requirement whose object is the record of activity is

applied	assigned to V&A in every Pillar.

REQ-27 Agent behavioral baseline and anomaly detection

Field	Content
Control objective	Establish behavioral baselines, detect goal drift, abnormal tool use, privilege escalation, and cross-agent anomalies, and trigger isolation or kill-switch responses when necessary.
Sources	S2, S9, R18, R19
Threat area	ASI-1, ASI-10
Pillar(s)	Identity; Devices & Runtime Environments; Networks; Applications, Workloads & Agent Actions; Data
Function(s)	ID-6, DE-5, NW-4, AW-9, DA-7
Assignment rationale	Behavioural baselines and anomaly detection are applied to the activity of every Pillar and are therefore realised through the V&A slot of each.
Boundary rule applied	Cross-cutting rule: as above.

REQ-28 Autonomy level management and deployment approval

Field	Content
Control objective	Require security review, approval of the Autonomy Level, and assignment of an accountable owner before deployment, and adjust autonomy according to trust and operational history.
Sources	S5, S6, S3, S2
Threat area	ASI-3, ASI-10
Pillar(s)	Applications, Workloads & Agent Actions; Identity; Devices & Runtime Environments; Networks; Data
Function(s)	AW-4, ID-8, DE-7, NW-6, AW-11, DA-9
Assignment rationale	Autonomy approval is a policy decision taken before deployment, so it is assigned to the Governance slot of every Pillar; the pre-deployment testing it depends on is a distinct control point and is assigned to AW-4.
Boundary rule applied	Cross-cutting rule: a requirement whose object is policy is assigned to Governance in every Pillar.

REQ-29 HITL optimization and approval fatigue prevention

Field	Content
Control objective	Prioritize human review by risk, automate low-risk tasks, and focus oversight on high-risk actions to reduce approval fatigue and automation bias.
Sources	S9, S2
Threat area	ASI-9
Pillar(s)	Identity; Devices & Runtime Environments; Networks; Applications, Workloads & Agent Actions; Data
Function(s)	ID-8, DE-7, NW-6, AW-11, DA-9
Assignment rationale	HITL prioritisation is a policy about how human oversight is allocated, so it is assigned to the Governance slot of every Pillar.
Boundary rule applied	Cross-cutting rule: as above.

REQ-30 Multi-agent pipeline trust management

Field	Content
Control objective	Monitor each agent's actions and trust state, and suspend the pipeline or isolate an agent when anomalous behavior or trust degradation is detected.
Sources	S2, S6, R18, R13
Threat area	ASI-8, ASI-10
Pillar(s)	Identity; Devices & Runtime Environments; Networks; Applications, Workloads & Agent Actions; Data
Function(s)	ID-7, DE-6, NW-5, AW-10, DA-8
Assignment rationale	Pipeline suspension and agent isolation are automated responses to a detected trust condition, so the requirement is assigned to the A&O slot of every Pillar.
Boundary rule applied	Cross-cutting rule: a requirement whose object is the automated response itself is assigned to A&O in every Pillar.

REQ-31 Agent lifecycle governance

Field	Content
Control objective	Govern agent creation, deployment, operation, and retirement, conduct periodic reviews, and verify the deletion of identities, credentials, memory, and delegation relationships at retirement.
Sources	S6, S5, S3
Threat area	ASI-3, ASI-10
Pillar(s)	Identity; Devices & Runtime Environments; Networks; Applications, Workloads & Agent Actions; Data
Function(s)	ID-8, DE-7, NW-6, AW-11, DA-9
Assignment rationale	Lifecycle governance sets policy for creation, operation, and retirement across all Pillars, so it is assigned to the Governance slot of each.
Boundary rule applied	Cross-cutting rule: as above.

The totals below exceed thirty-one because some requirements must be implemented at distinct control points in more than one Pillar.

Pillar	Requirements assigned
Identity	12
Devices & Runtime Environments	9
Networks	11
Applications, Workloads & Agent Actions	15
Data	12

4. Source Traceability

The table below lists the sources that contributed directly to one or more requirement-level control objectives. “Ref.” is the reference number used in the article, and “Key” is the identifier used throughout the coding record. Other sources cited in the article support the literature review, gap analysis, research method, or the domain example in Section 4.6, but do not serve as direct grounds for a security requirement and are therefore omitted here.

#	Source	Type	Requirements grounded
1	OWASP(2025)	Official document	REQ-01, REQ-02, REQ-04, REQ-05, REQ-06, REQ-08, REQ-09, REQ-10, REQ-11, REQ-12, REQ-13, REQ-14, REQ-15, REQ-16, REQ-18, REQ-19, REQ-20, REQ-21, REQ-22, REQ-23, REQ-25, REQ-26, REQ-27, REQ-28, REQ-29, REQ-30
2	Anthropic(2026)	Official document	REQ-01, REQ-03, REQ-07, REQ-28, REQ-31
4	Dehghantanha and Homayoun(2026)	Academic paper	REQ-19, REQ-27
6	Huang et al.(2026)	Academic paper	REQ-01, REQ-02
7	Liu et al.(2025)	Academic paper	REQ-24
9	Raza et al.(2026)	Academic paper	REQ-30
10	Wang et al.(2025)	Academic paper	REQ-12
13	CISA et al.(2026)	Official document	REQ-14, REQ-17, REQ-23, REQ-24, REQ-26, REQ-28, REQ-31
14	CSA(2026)	Official document	REQ-09, REQ-28, REQ-30, REQ-31
17	El Helou et al.(2026)	Academic paper	REQ-15, REQ-16
18	Goswami(2025)	Academic paper	REQ-03, REQ-04
19	Schroeder de Witt(2025)	Academic paper	REQ-27, REQ-30
20	Zhou et al.(2026)	Academic paper	REQ-21, REQ-22, REQ-23, REQ-24
21	Christodorescu et al.(2026)	Academic paper	REQ-14
22	OWASP(2025)	Official document	REQ-02, REQ-03, REQ-04, REQ-05, REQ-06, REQ-07, REQ-08, REQ-09, REQ-10, REQ-11, REQ-12, REQ-13, REQ-15, REQ-16, REQ-17, REQ-18, REQ-19, REQ-20, REQ-21, REQ-22, REQ-23, REQ-24, REQ-25, REQ-26, REQ-27, REQ-29
25	Narajala et al.(2026)	Academic paper	REQ-08
26	Peyrano(2026)	Academic paper	REQ-16
27	Rajagopalan and Rao(2026)	Academic paper	REQ-05
28	Huang et al.(2025)	Academic	REQ-10, REQ-18

		paper	
29	Maiti(2026)	Academic paper	REQ-06, REQ-07
30	Valente and Zalewski(2026)	Academic paper	REQ-14
